# Autosomal recessive cerebellar ataxias: a diagnostic classification approach according to ocular features

**DOI:** 10.3389/fnint.2023.1275794

**Published:** 2024-02-07

**Authors:** Diego Lopergolo, Francesca Rosini, Elena Pretegiani, Alessia Bargagli, Valeria Serchi, Alessandra Rufa

**Affiliations:** ^1^Department of Medicine, Surgery and Neurosciences, University of Siena, Siena, Italy; ^2^UOC Neurologia e Malattie Neurometaboliche, Azienda Ospedaliero-Universitaria Senese, Siena, Italy; ^3^UOC Stroke Unit, Department of Emergenza-Urgenza, Azienda Ospedaliero-Universitaria Senese, Siena, Italy; ^4^Unit of Neurology, Centre Hospitalier Universitaire Vaudoise Lausanne, Unit of Neurology and Cognitive Neurorehabilitation, Universitary Hospital of Fribourg, Fribourg, Switzerland; ^5^Evalab-Neurosense, Department of Medicine Surgery and Neuroscience, University of Siena, Siena, Italy

**Keywords:** autosomal recessive cerebellar ataxias, optical coherence tomography, eye-tracking, eye movements, artificial intelligence

## Abstract

Autosomal recessive cerebellar ataxias (ARCAs) are a heterogeneous group of neurodegenerative disorders affecting primarily the cerebellum and/or its afferent tracts, often accompanied by damage of other neurological or extra-neurological systems. Due to the overlap of clinical presentation among ARCAs and the variety of hereditary, acquired, and reversible etiologies that can determine cerebellar dysfunction, the differential diagnosis is challenging, but also urgent considering the ongoing development of promising target therapies. The examination of afferent and efferent visual system may provide neurophysiological and structural information related to cerebellar dysfunction and neurodegeneration thus allowing a possible diagnostic classification approach according to ocular features. While optic coherence tomography (OCT) is applied for the parametrization of the optic nerve and macular area, the eye movements analysis relies on a wide range of eye-tracker devices and the application of machine-learning techniques. We discuss the results of clinical and eye-tracking oculomotor examination, the OCT findings and some advancing of computer science in ARCAs thus providing evidence sustaining the identification of robust eye parameters as possible markers of ARCAs.

## Introduction

Autosomal recessive cerebellar ataxias (ARCAs) are a heterogeneous group of neurodegenerative disorders affecting primarily the cerebellum and/or its afferent tracts. ARCAs are often accompanied by damage of other neurological (e.g., corticospinal tracts, basal ganglia, vestibular and visual sensory systems, and peripheral nerves) or extra-neurological systems (e.g., muscle, heart, gastrointestinal tract, glands Rossi et al., 2018).

However, a variety of hereditary, acquired, and reversible etiologies can cause cerebellar dysfunction, leading to ataxia symptoms (Beaudin et al., 2019). Thus, to date, the diagnosis of ARCAs remains challenging, particularly in adult-onset sporadic cases. Remarkable advances have been achieved during the least decades in the molecular and pathologic characterization of mechanisms underlying ARCAs, opening up new unexpected diagnostic and therapeutic perspectives (Salem et al., 2023). Indeed, promising target therapies for some ataxias are at advanced development stages and the identification of quantitative markers of cerebellar function has become fundamental for a deep phenotyping and to precisely define possible outcome measures in clinical trials.

**Figure 1:**
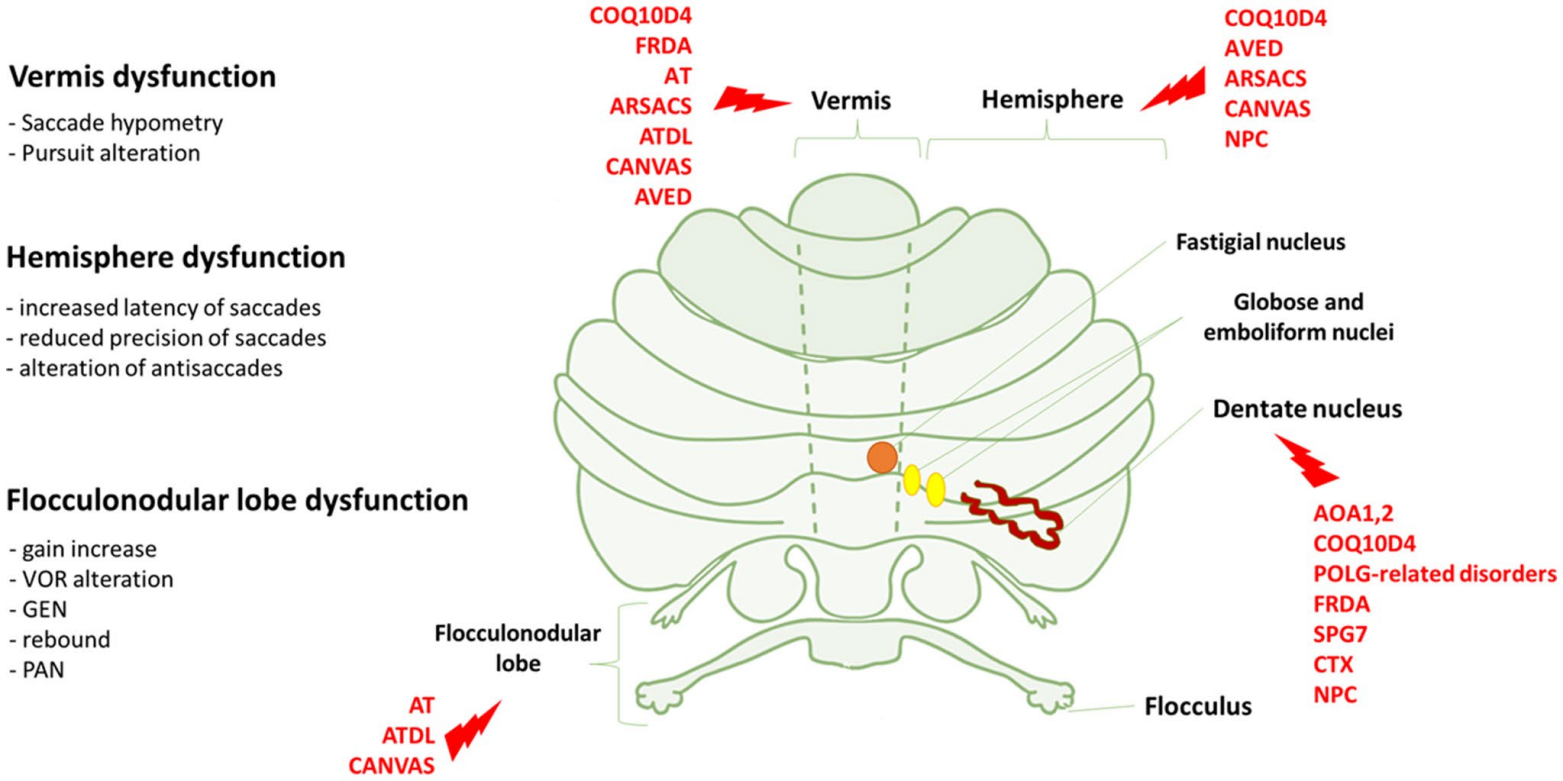
Eye movements dysfunction and genetic disorders associated to specific cerebellar areas. On the left, we reported the association between the dysfunction of vermis, hemisphere and flocculonodular lobe and the relative eye movements impairment. On the right, cerebellar regions whose dysfunctions are mostly associated with the genetic disorders indicated in red. FRDA, Freiderich’s ataxia; SPG7, Spastic Paraplegia 7; ARSACS, Autosomal Recessive Spastic Ataxia of Charlevoix-Saguenay; AT, Ataxia-Teleangiectasia; ATLD, ataxia-teleangiectasia like disorder; AOA1, ataxia with oculomotor Apraxia type 1; AOA2, Ataxia with Oculomotor Apraxia type 2; CTX, Cerebrotendinous Xanthomatosis; NPC, Nieman-Pick disease type C; AVED, ataxia with Vitamin E deficiency; COQ10D4, Coenzyme Q10 deficiency-4.

**Figure 2:**
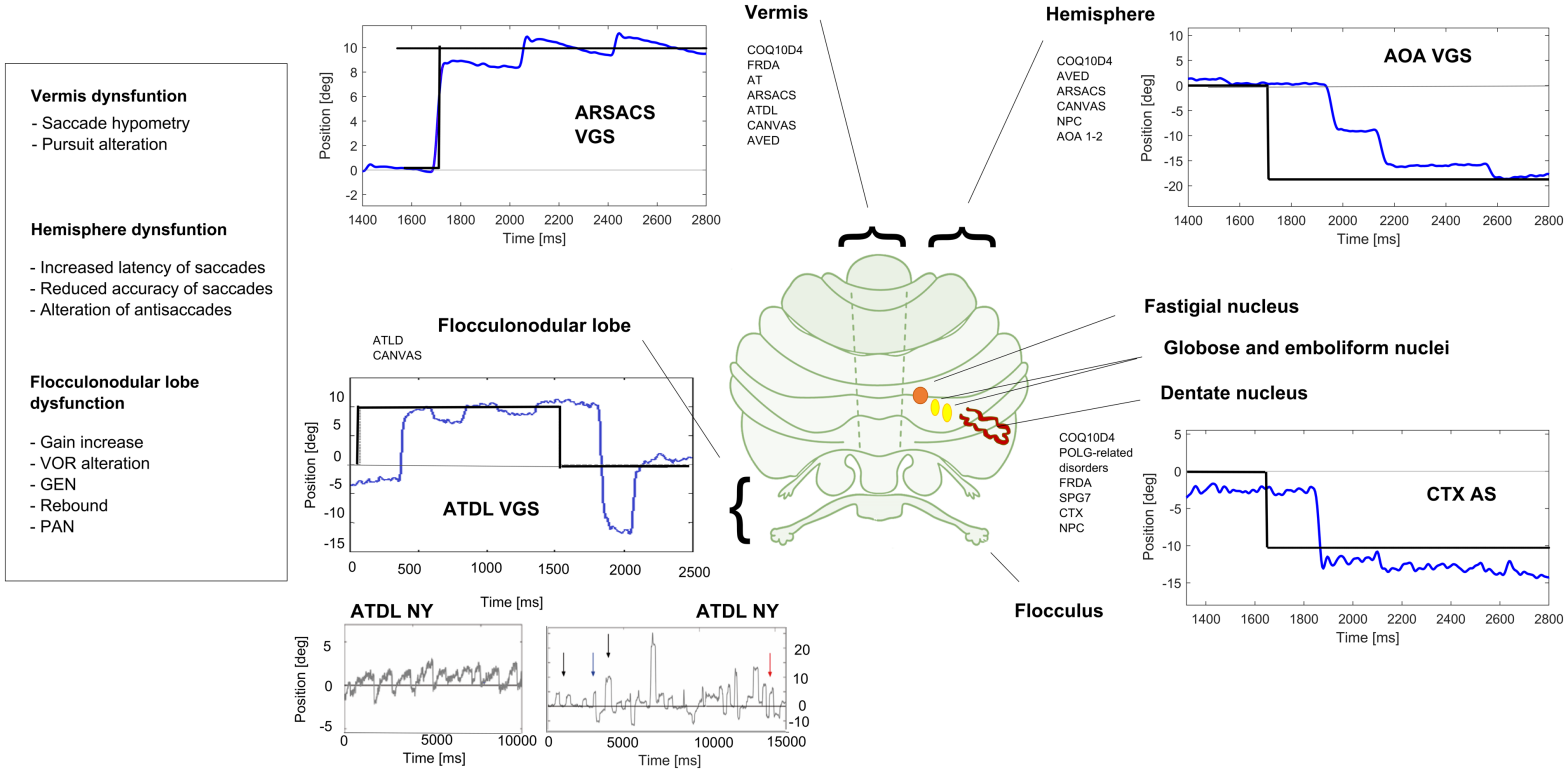
Eye movements in different cerebellar diseases recorded at EVA-Neurosense Lab (University of Siena). The prevalent oculomotor finding of each disease is associated to the mainly affected cerebellar region. ARSACS: VGS, hypometric vertical (8°) visually guided saccades, due to involvement of the oculomotor vermis. Gaze evoked ny is also evident, suggesting a loss of cerebellar neural integrator. AOA2: VGS, increased latency and multistep pattern of horizontal visually guided saccades (18°) due to hemispheric involvement. ATLD: VGS, dysmetric horizontal visually guided saccades (10°; undershooting and overshooting the target), first panel; ATLD Ny, rebound ny, second panel; ATLD: saccadic intrusion (SI), different amplitude and shape of high frequency saccadic intrusions, third panel. CTX AS, erroneous antisaccades (the movement is erroneously performed in the direction of the target instead in the opposite direction) saccades are hypometric. Axis: vertical (Y) indicates eye position in degrees; horizontal (X) indicates time in ms. FRDA, Freiderich’s ataxia; SPG7, Spastic Paraplegia 7; ARSACS, Autosomal Recessive Spastic Ataxia of Charlevoix-Saguenay; AT, Ataxia-Teleangiectasia; ATLD, ataxia-teleangiectasia like disorder; AOA1, ataxia with oculomotor Apraxia type 1; AOA2, Ataxia with Oculomotor Apraxia type 2; CTX, Cerebrotendinous Xanthomatosis; NPC, Nieman-Pick disease type C; AVED, ataxia with Vitamin E deficiency; COQ10D4, Coenzyme Q10 deficiency-4.

The examination of the afferent (retinal nerve fiber layer, ganglion cells) and efferent (fixation, saccades, pursuit, VOR) visual systems may represent a relevant source of neurophysiological and structural findings to study cerebellar dysfunction and neurodegeneration in ARCAs. This is well underlined by a recent consensus paper indicating that the study of eye movements is highly capable of localizing the anatomical lesion and is useful for the diagnosis and follow-up of patients (Garces et al., 2023). In this respect, various classes of eye movements have been associated to specific anatomic and functional regions of the cerebellum, as well as neural networks between these regions and extracerebellar regions (Leigh and Zee, 2015; Figures 1, 2). Moreover, the diffusion of new, low cost, low impact and easy to use eye trackers together with the application of machine learning techniques supported by Artificial Intelligence (AI) simplified the study of the oculomotor system (Stoean et al., 2020; Li et al., 2021). Collecting big oculomotor data from many cerebellar patients, would lead to the identification of a few specific oculo-motor indicators of cerebellar and extracerebellar functions (Cole et al., 2021).

Another important source to investigate the neurodegeneration in ARCAs comes from the parametrization of the optic nerve and macular areas by the Optical Coherence Tomography (OCT; Biousse et al., 2020). The assessment of the retinal thickness at the macular and optic nerve levels, measuring, respectively, the density of ganglion cells (GC) and retinal nerve fiber layers (RNFL), is often included in protocols for the phenotypic characterization of ARCAs and, in particular, for follow-up studies of Friederich’s ataxia (FRDA), Spastic Paraplegia 7 (SPG7) and Spastic Ataxia of Cherlevoix-Saguenay type (ARSACS).

In mitochondrial optic neuropathies OCT has proven to be a valuable tool with high diagnostic and prognostic value, also predicting the involvement of the fellow eye (Asanad et al., 2019). Notably, mitochondrial pathogenesis characterizes a significant subgroup of ARCAs, and such as FRDA, POLG-related ataxia, ARSACS, and SPG7, in which mutations of nuclear genes directly affect mitochondrial proteins, but also other forms, such as Ataxia-Teleangiectasia (AT), Ataxia-Teleangiectasia Like disease (ATLD), Ataxia with Ocular Apraxia 1 and 2 (AOA1, AOA2), Spinocerebellar ataxia with axonal neuropathy1 (SCAN 1), in which mitochondrial functions are also indirectly compromised.

In this paper we suggest the use of some robust eye findings as possible markers in the parametrization of ARCAs. We firstly resume the clinical ocular findings in different types of ARCAs thus providing a possible diagnostic classification approach according to ocular features deriving from the study of eye movements. Secondly, we discuss the quantification of the GC and RNFL thickness as a marker of neurodegeneration in hereditary ataxias. Lastly, we discuss how the advancement of computer science is making available and easier the study, diagnosis and analysis of ocular parameters.

## Diagnostic approach and common pathways underlying ARCAs

ARCAs are actually classified according to the most frequent ataxia-associated syndrome and to the underlying mutated gene (Beaudin et al., 2019; Table 1). However, the great phenotypic overlapping existing among ataxias sub-types categorized in different classes, increases the risk of missing the correct diagnosis and the appropriate treatments, even for the actually druggable types (i.e., Cerebrotendinous Xanthomatosis—CTX; Nieman-Pick—NPC; Ataxia with Vitamin E deficiency—AVED). To date, more than 90 autosomal-recessive disorders having ataxia as a predominant feature, as many, having ataxia as less predominant component (Rossi et al., 2018). This genetic pleiotropy is further augmented by the growing number of genes associated with both autosomal dominant ataxias and severe early-onset autosomal recessive ataxia syndromes (e.g., AFG3L2/SCA 28, SPTBN2/SCA5, ITPR1/SCA29, OPA1; Rossi et al., 2018). The advent of Next-Generation Sequencing (NGS) techniques has raised the diagnostic capacities and, at the same time, it has amplified the diagnostic complexity with ever-expanding pleiotropy of ARCAs genes (Galatolo et al., 2021). However, the wide used NGS approaches have the limitation of being unavailable in detecting pathological noncoding triplet expansions, such those occurring in FRDA or CANVAS, which are common ARCAs types. Furthermore, the diagnostic rate of NGS may reduce in case of mutations in new disease genes, deep intronic mutations, mutations in regulatory regions, gene dosage effects, or digenic modes of inheritance and epigenetic changes (Bonifert et al., 2014; Minnerop et al., 2017). Moreover, some genes may cause ataxia and spastic paraplegia suggesting a genetic and phenotypic continuum between Cerebellar ataxias (CA) and Hereditary Spastic paraplegias (Synofzik and Schüle, 2017). A stepwise diagnostic workout is currently recommended in ARCAs, starting from accurate clinical characterization, then testing a single gene when the suspicion is highly supported by the family history and presence of specific markers; alternatively it is suggested to test FRDA first, and then a NGS panel of genes or a whole exome sequencing.

**Table 1:** ARCAs classification by pathways involvement.

Pathways	Disease name (#OMIM)	Gene (protein)	Protein function	Inheritance (AD, autosomal dominant, AR, autosomal recessive)	Type of mutation	Gain of function/loss of function
**Mitochondrial pathways**						
	Friedreich ataxia (#229300)	*FXN* (frataxin)	Inner mitochondrial membrane protein regulating iron–sulfur cluster biogenesis	AR	96%: biallelic GAA repeat expansion within intron 1 (affected individuals have from 70 to more than 1,000 GAA triplets); 4%: monoallelic abnormally expanded GAA repeat and another intragenic pathogenic variant in the other allele	96% loss of function
	Mitochondrial recessive ataxia syndrome including SANDO and SCAE (#607459)	*POLG* (DNA polymerase subunit gamma-1)	The only DNA polymerase in humans that replicates and repairs mtDNA	AR	>95%: SNV or small intragenic deletions/insertions pathogenic variants; Very rare: deletion/duplication.	Loss of function
	Q10 deficiency-4 (#612016)	*COQ8A* or *ADCK3* (Coenzyme Q8A)	Coenzyme Q10 synthesis (Coenzyme Q10 is essential for proper functioning of the mitochondrial respiratory chain)	AR	SNV or small intragenic deletions/insertions pathogenic variants	“Ataxia-simplex phenotype,” more frequent with biallelic LOF variants; multisystemic phenotypes more frequently associated with missense variants (possible gain-of-function or dominant-negative effect)
	Spastic paraplegia 7 (# 607259)	*SPG7* (paraplegin)	Paraplegin-AFG3L2 complex is involved in mitochondrial protein maturation and degradation; its inactivation causes reduced mitochondrial complex I activity	AD, AR	>98%: SNV or small intragenic deletions/insertions pathogenic variants; <2%: deletion/duplication	Loss of function variants and missense variants
	Spastic ataxia 5, SPAX5 (# 614487)	*AFG3L2* (catalytic subunit of the m-AAA protease)	Paraplegin-AFG3L2 complex is involved in mitochondrial protein maturation and degradation. The m-AAA protease, an ATP-dependent proteolytic complex of the mitochondrial inner membrane that degrades misfolded proteins and regulates ribosome assembly	AR	Biallelic SNV or small intragenic deletions/insertions pathogenic variants	Loss of function
	Spinocerebellar Ataxia 28, SCA28 (# 610246)	*AFG3L2*	See above	AD	>99%: SNV or small intragenic deletions/insertions pathogenic variants; Extremely rare: monoallelic deletion/duplication	Loss of function variants and gain of function
	Spastic ataxia of the Charlevoix-Saguenay type, SACS, or ARSACS (# 270550)	*SACS* (sacsin)	Chaperon activities, controlling the microtubule balance or cell migration. Regulating the mitochondrial functions	AR	~95%: SNV or small intragenic deletions/insertions pathogenic variants; ~5%: deletion/duplication	Loss of function variants
**DNA repair/genome stability**						
	Ataxia-telangiectasia, AT (# 208900)	*ATM* (ATM)	Serine/threonine protein kinase of the phosphatidylinositol 3-kinase-related protein kinase (PIKK) family. In response to double strand breaks, ATM restores damaged sites, by phosphorylating numerous substrates.	AR	~90%: SNV or small intragenic deletions/insertions pathogenic variants; 1%–2%: deletion/duplication	Loss of function (prevalent) and gain of function
	Ataxia-telangiectasia like disorder, ATLD (# 604391)	*MRE11A* (MRE11A)	DNA repair (Mre11-Rad50-Nbs1 complex recruits ATM to the site of double strand breaks in nuclear DNA)	AR	n.a.	Loss of function
	Ataxia-oculomotor apraxia type 1, AOA1 (# 208920)	*APTX* (aprataxin)	Nuclear protein with hydrolase activities interacting with different pathways implicated in single-strand and double-strand repair mechanisms	AR	n.a.	Loss of function
	Ataxia with oculomotor apraxia type 2 (AOA2, SETX gene)	*SETX* (senataxin)	Ubiquitous high conservate RNA/DNA helicase that has been implicated in transcriptional regulation and the DNA damage response through resolution of R-loop structures	AR	~80%–92%: SNV or small intragenic deletions/insertions pathogenic variants; ~8%–20%: deletion/duplication	Loss of function
	Amyotrophic lateral sclerosis 4, ALS4 (# 602433)	*SETX* (senataxin)	See above	AD	See above	
	Cerebellar Ataxia Neuropathy Vestibular Areflexia Syndrome, CANVAS (# 614575)	*RFC1* (subunit of replication factor C)	Large subunit of replication factor C (RFC), a heteropentameric AAA+ protein complex associated with DNA synthesis during replication and repair	AR	biallelic pentanucleotide repeat expansion (AAGGG; 400 to 2,000 repeats). However, expansions may be AAAAG, AAAGG, AAGAG, AGAGG, ACAGG or AAGGG; imperfect repeats w/interruptions are also possible.	Unknown
**Complex lipids metabolism**						
	Niemann-Pick-type C disease, NPC (# 257220)	*NPC1* (95%) and *NPC2* (5%; NPC Intracellular Cholesterol Transporter 1 and 2)	Cellular trafficking of cholesterol and other lipids	AR	*NPC1*: 76% SNV or small intragenic deletions/insertions pathogenic variants; 22% deletion/duplication; 2% large deletions/duplications; *NPC2*: 88% SNV or small intragenic deletions/insertions pathogenic variants; 12% deletion/duplication	Loss of function
	Cerebrotendinous Xanthomatosis, CTX (#213700)	*CYP27A1* (mitochondrial sterol 27-hydroxylase)	Mitochondrial sterol 27-hydroxylase	AR	99%: SNV or small intragenic deletions/insertions pathogenic variants; 1%: deletion/duplication	Loss of function
	Ataxia with vitamin E deficiency, AVED (# 277460)	*TTPA* (Alpha Tocopherol Transfer Protein)	Protein that binds alpha-tocopherol, a form of vitamin E, and regulates vitamin E levels by transporting vitamin E between membrane vesicles and facilitating the secretion of vitamin E from hepatocytes to circulating lipoproteins.	AR	>90%: SNV or small intragenic deletions/insertions pathogenic variants	Loss of function

New mathematical methods have recently emerged for dealing with complexity of neurodegenerative disorders such as ARCAs; these methods are grounded on the application of the complex system frameworks, to assess interactions between intricate pathologic pathways underlying various *CA*. This approach allows us to identify a restrict number of pathologic nodes to which converge different metabolic pathways, that might even be shared across several ARCAs. This allows connecting genetically different diseases and focusing on molecular pathways shared across them, but also across other neurodegenerative diseases that may be targeted by common treatments and eventually monitored by the same biomarkers (Synofzik et al., 2019). However, despite the presence of such common pathways, the consequent pathogenetic cascade mechanisms may induce minor or greater impact on central nervous system structures, likely leading to variable ocular findings. In the cerebellum, this variability might be explained also by the presence of regions characterized by a different cells’ susceptibility due to a different glia-to-neuron ratio and consequent different energy demand (Emir et al., 2021). Three main pathological nodes to which converge different phenotypes can be identified in ARCAs (Table 1):

1. Mitochondrial metabolism
2. DNA repair/genome stability
3. Complex lipids metabolism

According to such three common pathways underlying ARCAs, we underly the presence of some robust eye findings that reinforce this new possible classification and can be used as possible markers in the parametrization of ARCAs.

## Oculomotor phenotypic characterization of ARCAs

### Mitochondrial pathways (FRDA, POLG, COQ8A, SPG7, SPAX5, and ARSACS)

Mitochondrial-induced high susceptibility to oxidative stress and free radicals’ toxicity and apoptosis mainly characterizes Friedreich ataxia (FRDA), POLG-related disorders and Coenzyme Q10 deficiency-4 (COQ10D4).

Among these, the most common genetically determined cerebellar ataxia is FRDA, a mixed ataxia with greater involvement of the posterior horns of the spinal cord and the dorsal root ganglia associated with progressive neurodegeneration of the cerebellum, cortico-spinal tracts, afferent visual pathway and systemic involvement (Table 2). FRDA is due to GAA repeat expansion within intron 1 of *FXN* gene resulting in decreased levels of Frataxin, which is an inner mitochondrial membrane protein regulating iron–sulfur (Fe-S) cluster biogenesis (Delatycki and Bidichandani, 2019). Thus, the primary event following frataxin deficiency seems to be the loss of Fe-S cluster biogenesis. Fe-S clusters are implicated in different cellular functions ranging from mitochondrial respiration, iron metabolism and translation to DNA repair. Frataxin inactivation is thus associated with increased cellular susceptibility to oxidative stress-toxicity (Martelli and Puccio, 2014) involving the nervous system and the visual pathway. Although in FRDA the papillomacular axonal system is not preferentially involved, in line with a progressive scattered loss of RGCs, complexes I, II and III are all involved, thus limiting possible compensatory mechanisms (Fortuna et al., 2009). FRDA patients usually show loss of large principal neurons and synaptic terminals in the dentate nucleus and possibly Purkinje cell injury (Kemp et al., 2016). This cascade mechanism may underlies also the various oculomotor abnormalities observed in FRDA: these include fixation instability with high rate of square wave jerks (SWJs), ocular flutter and gaze-evoked nystagmus, impaired smooth pursuit, increased latency of saccades and saccadic dysmetria (Furman et al., 1983; Hocking et al., 2010). Vestibulo-ocular reflexes and visual–vestibular interactions are also compromised (Fahey et al., 2008; Maudoux et al., 2020). Significant correlations have been reported between saccadic latency and disease severity as measured by the Friedreich Ataxia Rating Scale (FARS) and between fixation instability and age of disease onset. High order visual spatial abnormalities and deficits in disengaging fixation and executive functions have been reported in FRDA using gap-overlap, antisaccadic and memory guided paradigms (Rojas et al., 2021). Interestingly, the presentation and progression of compound heterozygotes FRDA patients may depend on the type of mutation on the second allele: loss-of-function mutations are associated with a significantly earlier age of onset; missense mutations on the second allele are often associated to optic neuropathy (Newman et al., 2023).

**Table 2:** Main oculomotor and fundoscopic findings.

	Impaired eye movements											Ophthalmoscopic and other ocular changes				
	Ptosis (drooping of the upper eyelid)	Nystagmus (rhythmic, involuntary, rapid, back and forth movement of the eyes)	Oscillopsia (illusion of an unstable visual world)	Vertical gaze impairment/palsy	Dysmetria	Increased latency/ocular apraxia (horizontal gaze failure due to deficits in starting voluntary/reactive eye movements)	Saccadic intrusions (conjugate small involuntary saccadic movements that disrupt visual fixation)	Impaired smooth pursuit (disturbance in maintain the line of sight on smoothly moving targets)	Impaired vestibulo ocular reflex (VOR is a reflex that maintains stable vision during rapid head rotations)	Extraocular muscles ophthalmoplegy	Strabismus (misaligned eyes)	Optic Atrophy	Optic nerve pallor	Juvenile cataract	Drusen (subretinal pigment epithelial deposit)	Retinitis pigmentosa
**Mitochondrial pathways**																
POLG	x	X			x					x		x				
SPG7	x	X		X						x						
ARSACS		X			x			x								
FRDA		X	x				x		x				x			
**DNA repair/genome stability**																
AT		X			x	x										
AOA1					x	X										
AOA2					x	X										
ATLD		x			x	x	x		x							
CANVAS		x	x						x							
**Complex lipid metabolism**																
NPC				X	x	x					x	x		x	x	
AVED											x					x

Mutations in the mitochondrial DNA *polymerase subunit gamma “POLG”*, responsible for the replication and repair of mitochondrial DNA, are increasing recognized causes of ataxia and peripheral neuropathy, variably associated to cerebellar or extracerebellar oculomotor changes (Wong et al., 2008). *POLG* mutations result in mitochondrial DNA depletion and/or multiple deletions (Rahman and Copeland, 2019) and are responsible of different phenotypes including *POLG related ataxia-neuropathy disorders (SANDO/MIRAS/arPEO plus)*.

*POLG* mutations are associated to premature mitochondrial dysfunction with increased oxidative stress and nuclear DNA damage, apoptosis activation, repression of regulators of mitochondrial biogenesis and cellular antioxidant response (Compton et al., 2011; Naess et al., 2011; Rouzier et al., 2014; Safdar et al., 2016). Secondary to these primary dysfunctions and similarly to what observed in FRDA, multiple pathways with differential cell vulnerability are involved (Synofzik et al., 2012). In *POLG*-mutated patients a severe myelin loss of the dentate and other deep cerebellar nuclei outflow tract was observed (Lax et al., 2012). In line with the expected muscular weakness associated to mitochondrial dysfunction, ophthalmoparesis and ptosis are the most frequently reported oculomotor finding in different POLG phenotypes (Rahman and Copeland, 2019). GEN and saccade dysmetria are also observed (unpublished data). Interestingly, hypertrophy of inferior olives has been described in several cases, and palatal tremor was reported in one case (Tilikete and Virginie, 2017), but no eye movements recording are available. Optic atrophy and pigmentary changes have been described in some cases (Rahman and Copeland, 2019).

Cerebellar ataxia with exercise intolerance characterizes biallelic *COQ8A* mutations leading to deficit in the CoQ10 biogenesis (Salviati et al., 2012; Caglayan et al., 2019). Fibroblast cell lines derived from mutated patients showed signs of oxidative stress and dysfunction in mitochondrial homeostasis (Cullen et al., 2016). Traschütz et al. (2020) studied genotype/phenotype correlations in patients with biallelic loss-of-function (LOF) and missense *COQ8A* mutations: they found that an “ataxia-simplex phenotype” was more frequent in patients with biallelic LOF variants, and conversely, missense variants having a possible dominant-negative effect were more often associated with multisystemic involvement beyond ataxia. The main phenotypic features of a large cohort of patients harboring biallelic *COQ8A* variants included early onset progressive cerebellar ataxia, seizures, cognitive impairment, hyperkinetic movement disorders and MR imaging of prevalent cerebellar vermal (100%) and hemisphere atrophy with dentate and pontine nuclei T2 hyperintensities (Horvath et al., 2012). Reported oculomotor abnormalities are ocular apraxia, slow saccades, and gaze palsy. Inconstant retinal pigmentary changes, cataracts, optic atrophy and hearing loss have also been reported (Traschütz et al., 2020). Unfortunately, eye movements are neither described nor recorded in this cohort even if according to neuroimaging of cerebellum and brainstem, the involvement of ocular motor system is likely.

Mitochondrial-related impairment of axonal transport mainly underlies spastic ataxias, particularly SPG7, in which ataxia and spasticity are often associated to impairment of optic pathway. Signs of cerebellar and extracerebellar oculomotor involvement and PEO are also described in these forms (de Bot et al., 2012; van Gassen et al., 2012; Pfeffer et al., 2014; Synofzik and Schüle, 2017). *SPG7* mutations affect the function of *Paraplegin*, an inner mitochondrial protein which assembles with homologous AFG3L2, to form the oligomeric mAAA protease complex (Casari et al., 1998; Giorgio and Roberto, 2018). The paraplegin-AFG3L2 complex is involved in mitochondrial protein maturation and degradation and its inactivation causes reduced mitochondrial complex I activity (Estrada-Cuzcano et al., 2017). Loss of AFG3L2 function is associated with autosomal recessive spastic ataxia 5 (SPAX5) and autosomal dominant spinocerebellar ataxia 28 (SCA28) with myopathy and CPEO (di Bella et al., 2010; Tulli et al., 2019).

Although SPG7 has been classically considered to show an autosomal recessive mode of inheritance, there is also evidence for autosomal dominant transmission in some families (Sánchez-Ferrero et al., 2013). The predominance of pyramidal signs is significantly associated to biallelic LOF variants rather than missense variants, suggesting that the loss of paraplegin function drives spasticity. Probably LOF of paraplegin still allows AFG3L2 to form functional oligomeric m-AAA protease and this could compensate for loss of paraplegin in the cerebellum because of the high AFG3L2 cerebellar expression (Coarelli et al., 2019). Moreover, nonsense variants on both alleles are associated to a more severe phenotype including ophthalmologic involvement. Multiple mitochondrial and extra-mitochondrial changes results from any dysfunctional *paraplegin*, including defective axonal transport particularly in synaptic terminals of long tract axons such as cortico-spinal and optic tracts with subsequent retrograde degeneration (Charif et al., 2020). Peripheral nerves are spared in SPG7, and this may differentiate SPG7 patients from ARSACS patients showing prominent peripheral sensory motor neuropathy and distal amyotrophy. SPG7 patients showed increased T2 signal from the dentate nucleus (Hewamadduma et al., 2018), in line with the postmortem data demonstrating neuronal loss in the dentate nucleus (Thal et al., 2015). The most frequently described oculomotor defect in SPG7 patients is the adult onset progressive external ophthalmoplegia and ptosis (CPEO; Klebe et al., 2012; van Gassen et al., 2012). However, cerebellar nystagmus and vertical gaze limitation with slow upward saccades and preserved VOR have also been reported. Supranuclear gaze palsy, seldom reported in SPG7, may be due to extension of neurodegeneration to the brainstem nuclei and pathways as demonstrated by pathologic studies (Thal et al., 2015).

*Sacsin* is involved in chaperon activities, controlling the microtubule balance or cell migration and it plays a crucial role in regulating the mitochondrial functions (Morani et al., 2019). Loss of function of *Sacsin* causes *autosomal recessive spastic ataxia of Charlevoix-Saguenay (ARSACS)*, the second most common ARCAs (Synofzik et al., 2013; Bradshaw et al., 2016; Vermeer et al., 2020). Sacsin deficiency determines an altered organization of intermediate filaments and consequently multiple mitochondrial dysfunctions involving mitochondrial fission (Girard et al., 2012), oxidative phosphorylation, and oxidative stress pathways (Criscuolo et al., 2015). This cascade of events may explain the presence of different clinical features partially reminiscent of mitochondrial disorders. Spastic ataxia is associated to peripheral mixed sensory motor neuropathy, progressive distal amyotrophy, mild intellectual disability, psychiatric symptoms, thickened optic disks and retinal architecture changes (Baets et al., 2010). Vermal and hemispheric cerebellar atrophy is a characteristic MRI finding, seldom associated to pontine hyperintensities, explaining the cerebellar origin of oculomotor and gaze holding changes. Cerebellar oculomotor deficits are clinically evident also in the atypical (only peripheral neuropathy) phenotypes of ARSACS (Vill et al., 2018) and include GEN and rebound nystagmus, saccadic pursuit and saccadic abnormalities. A supranuclear gaze palsy has also been reported in one patient (Stevens et al., 2013) while VOR changes have been not investigated.

### DNA repair/genome stability (AT, ATLD, AOA2, AOA1, AOA4, SCAN1, and RFC1-CANVAS)

Autosomal recessive ataxia with oculomotor apraxia (OMA), elevated levels of serum alpha-fetoprotein (AFP biomarker) and distinct mutations of genes implicated in DNA repair and/or transcription, define a group of early onset disorders including: ataxia-telangiectasia (*ATM* gene), ataxia-telangiectasia like disorder (*MERR11* gene) ataxia with oculomotor apraxia type 1 (AOA1, *APTX* gene) and type 2 (AOA2, *SETX* gene; Caldecott, 2003). These disorders, share common oculomotor abnormalities which demonstrate the functional failure of the cerebellar brainstem networks and vestibular system, and impairment of supranuclear input for the voluntary control of eye movement. Both oculomotor apraxia and long latency multistep saccades with oculocephalic dissociation are present in all disorders.

*Ataxia-Telangiectasia (AT)* and *Ataxia-Telangiectasia Like Disorder (ATLD)* are clinically similar conditions, with ATLD being very rare, less severe, and slowly progressive. AT is an early onset ARCAs due to mutations in *ATM*, encoding a serine/threonine protein kinase of the phosphatidylinositol 3-kinase-related protein kinase (PIKK) family (Gatti et al., 1999). In response to double strand breaks, ATM restores damaged sites (Fernandez-Capetillo et al., 2004). Also, *MRE11*, gene associated to ATLD, contributes to DNA repair, since this gene is part of the *Mre11-Rad50-Nbs1* complex (MRNcomplex). Both MRE11 and ATM respond to several stress and regulates multiple cellular pathways, explaining the multisystemic features observed in AT including degeneration of diverse neuronal systems (cerebellar, cortical, peripheral), increased sensitivity to ionizing radiation, cancer, immune deficiency, and diabetes (Bian et al., 2019). Conjunctival, auricular and buccal telangiectasias may be included in the AT phenotype. A recurrent MRI finding is the cerebellar atrophy mostly involving the vermis, in line with patients’ oculomotor presentation, and the anterior lobe, consistent with limb ataxia (Frismand et al., 2013). Cerebellar syndrome is progressively associated to other hyperkinetic disorders such as dystonia, chorea and peripheral sensory-motor axonal neuropathy (Stewart et al., 1999). Oculomotor abnormalities in AT and ATLD patients are mostly characterized by deficit in saccadic initiation and metrics, fixation, gaze holding, pursuits and VOR with oculocephalic incoordination (Palmeri et al., 2013; Federighi et al., 2017). Patients may show long latency reflexive saccades and even longer or absent volitional saccades, requiring a head thrust and blinking to, respectively, force eye deviation and reset eye position to allow the eyes to finally move into the desired position (Zee et al., 1977; Baloh, 1978). Saccades are hypometric or interrupted, with a characteristic stepwise gaze shift (Tang and Shaikh, 2019). Fixation is affected by spontaneous nystagmus (horizontal and vertical), slow drifts, periodic alternating nystagmus, and saccadic intrusions ranging from SWJ saccadic intrusions to micro-or macro saccadic oscillations and flutter (Lewis et al., 1999; Shaikh et al., 2009, 2011).

*AOA1 and AOA2* share similar oculomotor findings and clinical/MRI features, with AOA1 patients having early-onset slowly progressive cerebellar ataxia associated to axonal neuropathy, chorea, dystonia (which gradually attenuate), optional hypoalbuminemia, hypercholesterolemia and normal to slight increase of alpha-fetoprotein (Anheim et al., 2009). The loss of the normal hypointensity in the dentate nucleus was suggested as a highly sensitive and specific biomarker (Ronsin et al., 2019). In AOA1, *APTX* mutations lead to dysfunctional *aprataxin* which is a nuclear protein implicated in single-strand and double-strand repair mechanisms (Date et al., 2001; Le Ber et al., 2003). Cerebellar ataxia and axonal neuropathy develop later (second decade of life) in AOA2 patients, as well as pyramidal signs, dystonia, and chorea. AOA2 is due to dysfunctional *Senataxin* which is a ubiquitous high conservate RNA/DNA helicase implicated in transcriptional regulation and DNA damage response through resolution of R-loop structures (Richard and Rosonina, 2021). Mutations in *SETX* result in either of two distinct neurodegenerative disorders: recessive mutations are responsible for AOA2 (Moreira et al., 2004), while dominant mutations result in a juvenile form of amyotrophic lateral sclerosis (ALS) called ALS4 (Bennett and La Spada, 2021). A study of 90 individuals with *AOA2* found that pathogenic missense variants in the helicase domain caused less severe AOA2 phenotypes than missense variants outside of this domain, or deletions, or truncating variants. However, individuals with pathogenic truncating or missense variants outside of the helicase domain had a lower frequency of pyramidal signs—a finding that may reflect masking of pyramidal signs by severe motor neuropathy (Anheim et al., 2009).

Cerebellar-based oculomotor changes are common to both AOAs, including OMA (long latency hypometric saccades) and excessive blinking, which is reported in around 50% of patients; fixation instability with SWJ, GEN, downbeat and rebound Ny, saccadic pursuit, hypermetric saccades and staircase saccades are also present. Reduced vertical and horizontal saccade velocity, supranuclear ophthalmoplegia, bilateral esotropia or monocular strabismus as well as marked deficits of voluntary saccades are more prominent in AOA2, where the age at onset and presence of occasional oculomotor apraxia were negatively correlated to disease progression; ophthalmoplegia (no strabismus) was positively correlated to the progression of neurological disability (Anheim et al., 2010). Head trust with oculo-cephalic dissociation also develops in both conditions. In a recent study, hypometric saccades with a staircase pattern and no increased latency or head trust have been suggested to be a more reliable sign of oculomotor apraxia (Ronsin et al., 2019; Bargagli et al., 2021).

*Cerebellar Ataxia Neuropathy Vestibular Areflexia Sindrome (CANVAS)* is a late onset familial or sporadic form of sensory ataxia caused by recessive biallelic pentanucleotide repeat expansion (AAGGG) in the *RFC1 gene* which encodes the large subunit of replication factor C (RFC), a heteropentameric AAA+ protein complex associated with DNA synthesis during replication and repair. The expansion sizes vary from 400 to 2,000 repeats in affected patients (Cortese et al., 2019). The core CANVAS features are sensory neuronopathy, cerebellar ataxia, and a deficit of visually vestibulo-ocular reflex. Chronic spasmodic cough, oculomotor abnormalities usually including cerebellar ny and autonomic symptoms are also common (Szmulewicz et al., 2016). MRI shows cerebellar atrophy involving the vermis and hemispheres with Crus 1 (Traschütz et al., 2021). Oculomotor signs of vestibulocerebellar involvement are common in CANVAS, with progressive oscillopsia which can be permanent and mainly due to bilateral vestibular impairment. Nystagmus can be GEN, or downbeat, or combined; fixation instability both during head rotation, optokinetic ny, and during pursuit, and impaired vestibulo-ocular reflex and/or visuo-vestibular ocular reflex have been well described (Terryn et al., 2020).

Sensory ataxia and severe peripheral sensory neuropathy is common to CANVAS, FRDA and POLG; however, ocular findings allow to distinguish these three entities. Vestibular areflexia, oscillopsia and Ny, although present in both CANVAS and FRDA, are more severe in the former, while in FRDA saccadic intrusions are more evident. Moreover, optic nerve pallor is typical of FRDA (Dupré et al., 2021). Ptosis, ophthalmoparesis, saccade dynamic and metric changes are typical of POLG.

### Complex Lipids metabolism (NPC, CYP27A1 CTX, AVED)

*Complex Lipids metabolism disorders (NPC, CYP27A1 CTX, AVED)* includes rare conditions in which cerebellar ataxia is associated to the involvement of other cerebral structures and extra-brain organs. Different extent of oculomotor system, retina and optic nerve involvement is common. *Nieman-Pick-type C disease (NPC) i*s a rare, autosomal recessive, neuro-visceral disorder caused by mutations in either the *NPC1* (95%) or *NPC2* (5%) genes (Naureckiene et al., 2000; Patterson, 2020), both involved in the cellular trafficking of cholesterol and other lipids. Mutations result in the accumulation in multiple tissues of non-esterified cholesterol, sphingolipids, sphingomyelin, and phospholipids that accumulate primarily in the central nervous system, causing the neurological manifestations (Imrie et al., 2007). The metabolic pathway of the glycosphingolipids biosynthesis is the target of Miglustat, one of the approved treatments of NPC (Patterson et al., 2017). NPC ranges from a neonatal, rapidly fatal disorder to an adult-onset variant, characterized by cerebellar ataxia, dystonia or other movement disorders that may occur with atypical schizophrenia or early-onset psychosis and early onset cognitive decline, including deficits in attention, language, and executive functions. A pattern of fronto-temporal dementia may also be present. Brain MRI shows cerebellar atrophy with involvement of cerebellar cortex and dentate nucleus (Walterfang et al., 2012; Chiba et al., 2014). Vertical supranuclear gaze palsy is the hallmark of the disorder, starting with a progressive slowing of vertical saccades which assume a typical curved trajectory. Vertical saccades are a marker of disease progression; they have been used in clinical trials and are recommended for testing the effect of Miglustat. *Cerebrotendinous Xanthomatosis (CTX)* is a rare inherited lipid storage disorder caused by deficiency of mitochondrial sterol 27-hydroxylase, related to mutations in the *CYP27A1* gene (Koyama et al., 2021; Federico and Gallus, 2022), ultimately leading to an increase of plasma and tissue cholestanol levels (Salen and Steiner, 2017). The clinical symptomatology includes neonatal jaundice or cholestasis, diarrhea, tendon xanthomas, osteoporosis, coronary heart disease, juvenile cataracts, pulmonary involvement, progressive neuropsychiatric disturbances such as intellectual disability and dementia, psychiatric symptoms, pyramidal, extrapyramidal and cerebellar signs, and seizures (Mignarri et al., 2014; Salen and Steiner, 2017). Early replacement therapy with chenodeoxycholic acid may improve or even prevent CTX symptomatology (Stelten et al., 2019).

At neuroimaging examination, the most distinctive findings are represented by MRI hyperintensities in FLAIR or T2-weighted images in the dentate nuclei and cerebellar white matter. Juvenile cataract is an early peculiar feature (Khan et al., 2013; Tibrewal et al., 2017; Freedman et al., 2019). Electron microscopy of the cataract in a CTX patient disclosed membranous structures with vacuoles containing lipid materials. Replacement of cholesterol with cholestanol, leading to an impairment of lens function and alteration of cell membrane permeability, is considered the pathological substrate of cataracts in CTX (McKenna et al., 1990; Dotti et al., 2001). Optic disc atrophy and optic nerve abnormalities have been described in CTX patients (Van Bogaert et al., 1937; Guillain et al., 1942; Cruysberg et al., 1995; Dotti et al., 2001) and attributed to a defect in peripheral and central myelin synthesis related to the replacement of cholesterol with cholestanol. Other ophthalmic findings comprise retinal senescence, drusen, changes of retinal pigmented epitelium cells (Dotti et al., 2001), xanthelasma (Cruysberg et al., 1995), proptosis due to orbital xanthoma (Morgan et al., 1989), increased content of sterol in the extraocular muscles (Salen, 1971), bilateral exophthalmos (Kuriyama et al., 1991), acquired type II red-green defect of Verriest (Cruysberg et al., 1995). Efferent visual system abnormalities in CTX include gaze-evoked nystagmus and exotropia (Cruysberg et al., 1995). Saccades are accurate with normal main sequence relationship, but CTX patients tend to execute more frequent multistep saccades and directional errors during the antisaccade task than controls. Increased saccadic latency, impaired precision, increased directional errors are characteristic of CTX patients with involvement of dentate nuclei at brain MRI (Rosini et al., 2017).

*Ataxia with vitamin E deficiency (AVED)* is a rare progressive multisystem disorder usually occurring in young childhood. It is caused by omozygous or compound heterozygous mutations in the α-tocopherol transfer protein gene (*TTPA*) (Schuelke, 2023). Lack of gene product, a liver tocopherol-binding protein that incorporates alpha-tocopherol into nascent very-low-density lipoprotein (VLDL), causes vitamin E deficiency with subsequent systemic oxidative stress damage (Mariotti et al., 2004). Peculiar AVED features include progressive ataxia, dystonia, axonal neuropathy, pyramidal signs, and impaired proprioception; less frequently, scoliosis, tendon xanthomas and xanthelasmata, seizures, psychotic episodes, intellectual decline, and cardiomyopathy have been reported (Gabsi et al., 2001; Benomar et al., 2002; Becker et al., 2020). Neurological picture may resemble that of Friedreich’s ataxia. Brain MRI findings vary from preserved cerebellar volumes to vermian or hemispheric cerebellar atrophy (El Euch-Fayache et al., 2014). Purkinje cells deterioration is described in postmortem study (Mariotti et al., 2004), as well as retinal atrophy with massive accumulation of lipofuscin (Satya-Murti et al., 1986; Yokota et al., 2000). Retinitis pigmentosa is the most frequent neuro-ophthalmological feature (Yokota et al., 2000; El Euch-Fayache et al., 2014); in one case-report, macular degeneration has been reported (Iwasa et al., 2014). Head tremor is often encountered and can be associated with eye oscillations. Furthermore, nystagmus, oculomotor apraxia and exotropia were variably described in a cohort of Tunisian patients (El Euch-Fayache et al., 2014). Substitutive therapy with vitamin E can prevent symptoms in presymptomatic individuals and stabilize or ameliorate the clinical picture in symptomatic patients.

## Fundus photography and OCT parametrization of neurodegeneration in ARCAs

The pathologic process causing cerebellar neurodegeneration may also affect photoreceptors, ganglion cells, retinal vasculature, pigmented epithelium or axons of the optic nerve (Table 3). A first qualitative ophthalmoscopic assessment can be obtained by fundus photography which may help to compare the two eyes following ophthalmoscopic changes over time (Biousse et al., 2018). High quality fundoscopic images are also used for a quantitative assessment of retinal vasculature, retinal layers or optic nerve head structure and shape (Biousse and Newman, 2016; Panwar et al., 2016).

**Table 3:** Ocular coherence tomography assessment in the three most common ARCAs associated to mitochondrial pathway involvement.

	Ganglion cells	Retinal fibers	Optic nerve	Macula
ARSACS	Loss	Increased retinal peripapillary inner thickness	Progressive optic nerve atrophy	Macula microcysts, Foveal hypoplasia
			/	
FRDA	Loss	RNFL average thickness reduced	Progressive neurorim pallor	/
SPG7	Loss	RNFL average thickness reduced, > in temporal quadrant	Optic neuropathy (in rarely cases is the only manifestation)	/

Digital image processing is one of the most widely used computer vision technology in biomedical engineering and various digital techniques are used to analyze retinal deposits, exudates, and microvasculature and to measure geometric features such as vessel tortuosity, branching angles, branching coefficient, vessel diameter, and fractal dimension (Rajalakshmi et al., 2021). The extracted markers of fundus digital images, provide quantitative assessment of retinal topographic changes associated to various diseases (Kipli et al., 2018).

OCT is a widely used retinal imaging technique providing high resolution cross-sectional view of retinal layers and retinal thickness. Light reflectance principles underly spectral domain OCT (SD-OCT) and multicolor OCT (MC-OCT) technology, that makes the retina and optic nerve visible at a 5- to 6-nm resolution in an imaging process that requires only 2 min per eye (Minakaran et al., 2021). Combining structural and functional (visual) information, machine learning classification, and AI methods, allows us to elaborate digital markers of retinal and optic nerve degeneration or validate OCT intra or inter-eye morpho-functional thresholds (Pujari et al., 2021).

The further innovation introduced by MC-OCT is the use of 3 different, simultaneous laser light wavelengths to produce “fundus photography tomograms” of deep, mid, and inner retinal layers. MC-OCT uses ultrared (815 nm), green (518 nm), and blue (486 nm) lights to reflect the retina over almost the entire visible light spectrum. These different wavelengths create focused and detailed images especially of the retinal blood vessels. The assessment of retinal vessels by MC-OCT together with the retinal GC density and peripapillary nerve fiber layer (pRNFL) measurements (which reflects the safety of optic nerves, chiasm, and optic tracts) by spectral domain OCT, allow finding out *“in vivo”* optic nerve and retinal structural changes and the inter-eye differences in acquired or genetic optic neuropathies, including those encountered in ARCAs with relevant clinical and prognostic implications.

### RNFL and ganglion cells assessment

Optic neuropathy is frequently observed in ARCAs, particularly in those forms primarily related to damage of mitochondrial pathway such as *FRDA, SPG7, ARSACS*, where retinal GCs death is a specific target for mitochondrial mediated neurodegeneration in the retina (Newman et al., 2023).

Mitochondrial optic neuropathies are a relatively homogeneous group of visual disorders characterized by preferential involvement of small axons of the papillo-macular bundle, serving central vision, color vision and high spatial frequency contrast sensitivity (Carelli et al., 2004). The high vulnerability of central retinal GCs to mitochondrial damage is due to prevalent dependence of the intra-retinal axonal transport from mitochondrial metabolism. Differently from hereditary non-syndromic mitochondrial-related optic neuropathies such as LHON, the papillo-macular bundle is less affected at onset in *FRDA* and other mitochondrial related ARCAs, leaving visual acuity, contrast sensitivity and color vision, longer preserved than LHON. However, the advent of OCT has evidenced an early axonal damage and GCs loss in several ARCAs including FRDA. This technique also allows to monitor changes of retinal thickness over time supporting a role for these structural measures, as a marker of neurodegeneration in these diseases.

Neurorim pallor is clinically apparent in 30% of FRDA patients at the onset (Harding, 1981; Fortuna et al., 2009). Several studies (Fortuna et al., 2009; Noval et al., 2012; Seyer et al., 2013; Dağ et al., 2014) have evaluated RNFL and GC changes using OCT showing decreased average RNFL thickness, which was statistically significant in comparison to controls. Abnormal RNFL thinning in all quadrants has been reported, with a distinctive pattern of predominant involvement of superior quadrant, while macular and foveal thickness is generally preserved in these patients. Recently, FRDA was reported to be associated with the greatest degree of RNFL thinning in comparison to a range of other genetically determined ataxias. RNFL thickness in FRDA has been shown to correlate with neurological function and disability as measured with the International Cooperative Ataxia Rating Scale (ICARS). More recently, a correlation between the peripapillary RNFL and the Scale for Assessment and Rating of Ataxia (SARA) used to quantify disability was demonstrated by Parkinson et al. (2018) and Rojas et al. (2021). Another significant correlation has been found between thinning of the RNFL and frataxin protein levels, leading authors to suggest the use of these two measures as biomarkers in future clinical trial design (Thomas-Black et al., 2023).

Degenerative optic neuropathy is also reported in several families with SPG7; however, not all patients have reduced visual acuity. Optic atrophy occurred in 3/49 cases of SPG7 in a Dutch cohort; two SPG7 mutated sibs with optic atrophy manifested severe vision loss as the presenting symptom evolving to full blindness in the course of the illnesses. Post-mortem examination of one of these sibs disclosed severe atrophy affecting the optic nerves, optic chiasm and optic tracts with loss of axons, demyelination and astrogliosis (van Gassen et al., 2012). In accordance with these data, employing OCT, global RNFL thinning in individuals with SPG7 has been reported in several cohorts (Wiethoff et al., 2012). It has been shown that retinal fibers loss is more pronounced in the temporal quadrant (Bogdanova-Mihaylova et al., 2021). Patients with severe loss of RNFL and low visual acuity may have a more complex phenotype including intellectual disability, cognitive decline, limb spasticity and cerebellar ataxia (Wiethoff et al., 2012). In 65% of patients belonging to a large SPG7 family (Klebe et al., 2012), authors found the homozygous Ala510Val mutation associated with optic neuropathy, present in all patients. Moreover, the same authors showed a novel missense *SPG7* mutation in heterozygous state (Asp411Ala) as the cause of autosomal dominant optic neuropathy in a large family, indicating that some *SPG7* mutations can occasionally be dominantly inherited and be an uncommon cause of isolated optic neuropathy. *Paraplegin* is a metalloprotease known to cleave OPA protein 1 (a mitochondrial dynamin-related protein) into two active subunits, which locate in the inner mitochondrial membrane and regulate multiple mitochondrial functions, including fusion and fission (Ferreirinha et al., 2004; Martinelli and Rugarli, 2010). Abnormal *paraplegin* function may therefore result in a damage for GCs or optic nerve axons like those commonly found in non-syndromic mitochondrial optic neuropathy.

In ARSACS (Girard et al., 2012) the characteristic ophthalmic hallmark is an increased peripapillary inner retinal thickness, previously mis-characterized as hyper-myelinated nerve fiber layer. Conversely, visual loss is rare and unrelated to RNFL thickening (Garcia-Martin et al., 2013; Parkinson et al., 2018; Rezende Filho et al., 2021). OCT helped to clarify the characteristic modifications of the retinal layers in ARSACS showing retinal striations or folds with sawtooth appearance, foveal hypoplasia in 100% of patients, macular microcysts, papillomacular folds and thick peripapillary RNFL. Several studies have indicated that the thickness of all retinal layers is a distinctive pattern of ARSACS suggesting a role of the *SACS* gene in the development of the retina and optic nerve (Gazulla et al., 2011; Synofzik et al., 2013). *Sacsin* has been confirmed to be involved in regulating mitochondrial dynamics, in promoting neurofilament assembly or resolving their bundling accumulations and modulating interactions between cytoskeletal and synaptic adhesion proteins (Bradshaw et al., 2016; Romano et al., 2022).

## AI and parametrization of oculomotor data

Artificial intelligence (AI) is a branch of computer science seeking to simulate human intelligence in computers. AI is currently applied in medicine to accurately identify abnormalities in clinical, imaging or electrophysiological parameters and to distinguish individuals deviating from healthy controls. The term “artificial intelligence” includes machine learning and deep learning approaches. *Machine learning* is commonly described as “a field of study that gives computers the ability to learn without being explicitly programmed.” Characteristically, machine learning programs can modify the parameters of their algorithms by the exposure to more data, featuring in this way an adaptive response to the presented data and the ability to make predictions based on parameters related to their algorithms. *Deep learning* is a subset of machine learning based on artificial neural networks trying to mimic the complex computation capabilities, decision-making patterns, and neural connectivity typical of the human brain. Complex, multi-layered neural networks are assembled for allowing data to pass between nodes (like neurons) in highly connected ways, reaching increasingly non-linear transformation of the data.

Convolutional neural networks are capable of image recognition and classification; they are a crucial component of deep learning applications in medicine, especially in OCT imaging elaboration. The application of AI and deep learning techniques using OCT images has increased in recent years to assist clinicians in the diagnosis and management of neuroophthalmological diseases (Kapoor et al., 2019; Milea et al., 2020). Machine learning and deep learning are also applied in video-based eye-tracking technology using the existing front-facing cameras of smartphones and laptops to record eye movements. Machine learning models trained on eye movement features are then used to accurately identify eye abnormalities. This approach has been tested in ataxia patients where the algorithm detects saccade dysmetria (Azami et al., 2022) or pursuit changes in affected subjects (Chang et al., 2020) showing good performances (Azami et al., 2022).

## Conclusion

In conclusion, ocular features may be considered as ideal biomarkers for disease assessment in autosomal recessive cerebellar ataxias (ARCAs). In this perspective, the quantification of eye movements changes may help localizing the cerebellar-extracerebellar networks specifically involved in ARCAs; at the same time, the quantification of structural retinal changes by OCT provides a measure of neurodegeneration particularly in those forms related to mitochondrial pathway dysfunctions. Thus, the examination of visual system may drive a possible diagnostic classification approach according to ocular features since the three common pathways underlying ARCAs, i.e., mitochondrial metabolism, DNA repair/genome stability and complex lipid metabolism, show, respectively, peculiar pattern of visual system dysfunction.

The recent advances of AI, the use of sensors and other embedded devices is paving the way for real-life use of digital biomarkers; among these, the oculomotor markers and OCT cannot be missing in cerebellar ataxias.

## Author contributions

DL: Conceptualization, Writing – original draft, Writing – review & editing. FR: Writing – original draft, Writing – review & editing. EP: Writing – original draft, Writing – review & editing. AB: Writing – original draft, Writing – review & editing. VS: Writing – original draft, Writing – review & editing. AR: Conceptualization, Supervision, Writing – original draft, Writing – review & editing.

## Figures and Tables

**Table:** 

ARCAs	Autosomal Recessive Cerebellar Ataxias
VOR	Vestibulo-ocular reflex
AI	Artificial intelligence
OCT	Optical Coherence Tomography
RNFL	Retinal nerve fiber layer
FRDA	Freiderich’s ataxia
SPG7	Spastic Paraplegia 7
ARSACS	Autosomal Recessive Spastic Ataxia of Charlevoix-Saguenay
AT	Ataxia-Telangiectasia
ATLD	Ataxia-telangiectasia like disorder
AOA1	Ataxia with oculomotor Apraxia type 1
AOA2	Ataxia with Oculomotor Apraxia type 2
SCAN1	SpinoCerebellar Ataxia with Neuropathy1
CTX	Cerebrotendinous Xanthomatosis
NPC	Nieman-Pick disease type C
AVED	Ataxia with Vitamin E deficiency
NGS	Next-generation sequencing
CA	Cerebellar ataxias
COQ10D4	Coenzyme Q10 deficiency-4
SWJs	Square Wave Jerks
FARS	Friederich’s Ataxia Taring Scale
SANDO	Sensory Ataxic Neuropathy, Disartria and Ophthalmoparesis
MIRAS	Mithocondrial recessive Ataxia syndrome
PEO	Progressive Esternal Ophthalmoparesis
RFC	Replication Factor C
ICARS	International Cooperative Ataxia rating Scale
SARA	Scale for Assessment and Rating of Ataxia

## References

[ref1] Anheim M. Fleury M. Monga B. Laugel V. Chaigne D. Rodier G.. (2010). Epidemiological, clinical, Paraclinical and molecular study of a cohort of 102 patients affected with autosomal recessive progressive cerebellar ataxia from Alsace, eastern France: implications for clinical management. Neurogenetics 11, 1–12. doi: 10.1007/s10048-009-0196-y, PMID: 19440741

[ref2] Anheim M. Monga B. Fleury M. Charles P. Barbot C. Salih M.. (2009). Ataxia with Oculomotor apraxia type 2: clinical, biological and genotype/phenotype correlation study of a cohort of 90 patients. Brain 132, 2688–2698. doi: 10.1093/brain/awp211, PMID: 19696032

[ref3] Asanad S. Tian J. J. Frousiakis S. Jiang J. P. Kogachi K. Felix C. M.. (2019). Optical coherence tomography of the retinal ganglion cell complex in Leber’s hereditary optic neuropathy and dominant optic atrophy. Curr. Eye Res. 44, 638–644. doi: 10.1080/02713683.2019.1567792, PMID: 30649972

[ref4] Azami H. Chang Z. Arnold S. E. Sapiro G. Gupta A. S. (2022). Detection of Oculomotor Dysmetria from Mobile phone video of the horizontal saccades task using signal processing and machine learning approaches. IEEE Access 10, 34022–34031. doi: 10.1109/ACCESS.2022.3156964, PMID: 36339795 PMC9632643

[ref5] Baets J. Deconinck T. Smets K. Goossens D. van den Bergh P. Dahan K.. (2010). Mutations in SACS cause atypical and late-onset forms of ARSACS. Neurology 75, 1181–1188. doi: 10.1212/WNL.0b013e3181f4d86c, PMID: 20876471

[ref6] Baloh R. W. (1978). Internuclear Ophthalmoplegia. Arch. Neurol. 35:484. doi: 10.1001/archneur.1978.00500320004002 666604

[ref7] Bargagli A. Rosini F. Zanca D. Serchi V. Rufa A. (2021). Ataxia with Oculomotor apraxia type 2 (AOA2): an eye movement study of two siblings. Neurol. Sci. 42, 3039–3042. doi: 10.1007/s10072-021-05206-1, PMID: 33770309

[ref8] Beaudin M. Matilla-Dueñas A. Soong B.-W. Pedroso J. L. Barsottini O. G. Mitoma H.. (2019). The classification of autosomal recessive cerebellar ataxias: a consensus statement from the Society for Research on the cerebellum and ataxias task force. Cerebellum 18, 1098–1125. doi: 10.1007/s12311-019-01052-2, PMID: 31267374 PMC6867988

[ref9] Becker A. E. Vargas W. Pearson T. S. (2020). Ataxia with vitamin e deficiency may present with cervical dystonia. Tremor Other Hyperkin Movements 6:298. doi: 10.5334/tohm.298 PMC4884265 27274910

[ref10] Bennett C. L. La Spada A. R. (2021). SUMOylated Senataxin functions in genome stability, RNA degradation, and stress granule disassembly, and is linked with inherited ataxia and motor neuron disease. Mol Genet Genomic Med 9:e1745. doi: 10.1002/mgg3.1745, PMID: 34263556 PMC8683630

[ref11] Benomar A. Yahyaoui M. Meggouh F. Bouhouche A. Boutchich M. Bouslam N.. (2002). Clinical comparison between AVED patients with 744 Del a mutation and Friedreich ataxia with GAA expansion in 15 Moroccan families. J. Neurol. Sci. 198, 25–29. doi: 10.1016/S0022-510X(02)00057-6 12039660

[ref12] Bian L. Meng Y. Zhang M. Li D. (2019). MRE11-RAD50-NBS1 complex alterations and DNA damage response: implications for cancer treatment. Mol. Cancer 18:169. doi: 10.1186/s12943-019-1100-5, PMID: 31767017 PMC6878665

[ref13] Biousse V. Bruce B. B. Newman N. J. (2018). Ophthalmoscopy in the 21st century. Neurology 90, 167–175. doi: 10.1212/WNL.0000000000004868, PMID: 29273687 PMC5798658

[ref14] Biousse V. Newman N. J. (2016). Diagnosis and clinical features of common optic neuropathies. Lancet Neurol 15, 1355–1367. doi: 10.1016/S1474-4422(16)30237-X 27839652

[ref15] Biousse V. Newman N. J. Najjar R. P. Vasseneix C. Xu X. Ting D. S.. (2020). Optic disc classification by deep learning versus expert Neuro-ophthalmologists. Ann. Neurol. 88, 785–795. doi: 10.1002/ana.25839, PMID: 32621348

[ref16] Bogdanova-Mihaylova P. Plapp H. M. Chen H. Early A. Cassidy L. Walsh R. A.. (2021). Longitudinal assessment using optical coherence tomography in patients with Friedreich’s ataxia. Tomography 7, 915–931. doi: 10.3390/tomography7040076, PMID: 34941648 PMC8706975

[ref17] Bonifert T. Karle K. N. Tonagel F. Batra M. Wilhelm C. Theurer Y.. (2014). Pure and Syndromic optic atrophy explained by deep Intronic OPA1 mutations and an Intralocus modifier. Brain 137, 2164–2177. doi: 10.1093/brain/awu165, PMID: 24970096 PMC4107747

[ref18] Bradshaw T. Y. Romano L. E. L. Duncan E. J. Nethisinghe S. Abeti R. Michael G. J.. (2016). A reduction in Drp1-mediated fission compromises mitochondrial health in autosomal recessive spastic ataxia of Charlevoix Saguenay. Hum. Mol. Genet. 25, 3232–3244. doi: 10.1093/hmg/ddw173, PMID: 27288452 PMC5179924

[ref19] Caglayan A. O. Gumus H. Sandford E. Kubisiak T. L. Ma Q. Ozel A. B.. (2019). COQ4 mutation leads to childhood-onset ataxia improved by CoQ10 administration. Cerebellum 18, 665–669. doi: 10.1007/s12311-019-01011-x, PMID: 30847826 PMC6536000

[ref20] Caldecott K. W. (2003). DNA single-Strand break repair and Spinocerebellar ataxia. Cell 112, 7–10. doi: 10.1016/S0092-8674(02)01247-3 12526788

[ref21] Carelli V. Ross-Cisneros F. N. Sadun A. A. (2004). Mitochondrial dysfunction as a cause of optic neuropathies. Prog. Retin. Eye Res. 23, 53–89. doi: 10.1016/j.preteyeres.2003.10.003 14766317

[ref22] Casari G. de Fusco M. Ciarmatori S. Zeviani M. Mora M. Fernandez P.. (1998). Spastic paraplegia and OXPHOS impairment caused by mutations in Paraplegin, a nuclear-encoded mitochondrial Metalloprotease. Cell 93, 973–983. doi: 10.1016/S0092-8674(00)81203-9, PMID: 9635427

[ref23] Chang Z. Chen Z. Stephen C. D. Schmahmann J. D. Wu H.-T. Sapiro G.. (2020). Accurate detection of cerebellar smooth pursuit eye movement abnormalities via Mobile phone video and machine learning. Sci. Rep. 10:18641. doi: 10.1038/s41598-020-75661-x, PMID: 33122811 PMC7596555

[ref24] Charif M. Chevrollier A. Gueguen N. Bris C. Goudenège D. Desquiret-Dumas V.. (2020). Mutations in the M-AAA proteases AFG3L2 and SPG7 are causing isolated dominant optic atrophy. Neurology 6:428. doi: 10.1212/NXG.0000000000000428 PMC7251510 32548275

[ref25] Chiba Y. Komori H. Takei S. Hasegawa-Ishii S. Kawamura N. Adachi K.. (2014). Niemann-Pick disease type <scp>C</Scp> 1 predominantly involving the Frontotemporal region, with cortical and brainstem <scp>L</Scp> Ewy bodies: an autopsy case. Neuropathology 34, 49–57. doi: 10.1111/neup.12047, PMID: 23711246

[ref26] Coarelli G. Schule R. van de Warrenburg B. P. C. de Jonghe P. Ewenczyk C. Martinuzzi A.. (2019). Loss of Paraplegin drives spasticity rather than ataxia in a cohort of 241 patients with SPG7. Neurology 92, e2679–e2690. doi: 10.1212/WNL.0000000000007606, PMID: 31068484 PMC6556095

[ref27] Cole Z. J. Kuntzelman K. M. Dodd M. D. Johnson M. R. (2021). Convolutional neural networks can decode eye movement data: a black box approach to predicting task from eye movements. J. Vis. 21:9. doi: 10.1167/jov.21.7.9, PMID: 34264288 PMC8288051

[ref28] Compton A. G. Troedson C. Wilson M. Procopis P. G. Li F.-Y. Brundage E. K.. (2011). Application of oligonucleotide Array CGH in the detection of a large intragenic deletion in POLG associated with Alpers syndrome. Mitochondrion 11, 104–107. doi: 10.1016/j.mito.2010.07.012, PMID: 20708716

[ref29] Cortese A. Simone R. Sullivan R. Vandrovcova J. Tariq H. Yau W. Y.. (2019). Biallelic expansion of an Intronic repeat in RFC1 is a common cause of late-onset ataxia. Nat. Genet. 51, 649–658. doi: 10.1038/s41588-019-0372-4 30926972 PMC6709527

[ref30] Criscuolo C. Procaccini C. Meschini M. C. Cianflone A. Carbone R. Doccini S.. (2015). Powerhouse failure and oxidative damage in autosomal recessive spastic ataxia of Charlevoix-Saguenay. J. Neurol. 262, 2755–2763. doi: 10.1007/s00415-015-7911-4, PMID: 26530509

[ref31] Cruysberg J. R. M. Wevers R. A. van Engelen B. G. M. Pinckers A. van Spreeken A. Tolboom J. J. M. (1995). Ocular and systemic manifestations of Cerebrotendinous Xanthomatosis. Am J. Ophthalmol. 120, 597–604. doi: 10.1016/S0002-9394(14)72206-8, PMID: 7485361

[ref32] Cullen J. K. Abdul Murad N. Yeo A. McKenzie M. Ward M. Chong K. L.. (2016). AarF domain containing kinase 3 (ADCK3) mutant cells display signs of oxidative stress, defects in mitochondrial homeostasis and Lysosomal accumulation. PLoS One 11:e0148213. doi: 10.1371/journal.pone.0148213, PMID: 26866375 PMC4751082

[ref33] Dağ E. Örnek N. Örnek K. Erbahçeci-Timur I. E. (2014). Optical coherence tomography and visual field findings in patients with Friedreich ataxia. J. Neuroophthalmol. 34, 118–121. doi: 10.1097/WNO.0000000000000068, PMID: 24275983

[ref34] Date H. Onodera O. Tanaka H. Iwabuchi K. Uekawa K. Igarashi S.. (2001). Early-onset ataxia with ocular motor apraxia and Hypoalbuminemia is caused by mutations in a new HIT superfamily gene. Nat. Genet. 29, 184–188. doi: 10.1038/ng1001-184, PMID: 11586299

[ref35] de Bot S. T. Willemsen M. A. A. P. Vermeer S. Kremer H. P. H. van de Warrenburg B. P. C. (2012). Reviewing the genetic causes of spastic-ataxias. Neurology 79, 1507–1514. doi: 10.1212/WNL.0b013e31826d5fb0 23033504

[ref36] Delatycki M. B. Bidichandani S. I. (2019). Friedreich ataxia-pathogenesis and implications for therapies. Neurobiol. Dis. 132:104606. doi: 10.1016/j.nbd.2019.104606, PMID: 31494282

[ref37] di Bella D. Lazzaro F. Brusco A. Plumari M. Battaglia G. Pastore A.. (2010). Mutations in the mitochondrial protease gene AFG3L2 cause dominant hereditary ataxia SCA28. Nat. Genet. 42, 313–321. doi: 10.1038/ng.544, PMID: 20208537

[ref38] Dotti M. T. Rufa A. Federico A. (2001). Cerebrotendinous Xanthomatosis: heterogeneity of clinical phenotype with evidence of previously Undescribed ophthalmological findings. J. Inherit. Metab. Dis. 24, 696–706. doi: 10.1023/A:1012981019336, PMID: 11804206

[ref39] Dupré M. Hermann R. Froment Tilikete C. (2021). Update on cerebellar ataxia with neuropathy and bilateral vestibular Areflexia syndrome (CANVAS). Cerebellum 20, 687–700. doi: 10.1007/s12311-020-01192-w, PMID: 33011895 PMC8629873

[ref40] El Euch-Fayache G. Bouhlal Y. Amouri R. Feki M. Hentati F. (2014). Molecular, clinical and peripheral neuropathy study of Tunisian patients with ataxia with vitamin E deficiency. Brain 137, 402–410. doi: 10.1093/brain/awt339, PMID: 24369383

[ref41] Emir U. E. Sood J. Chiew M. Thomas M. A. Lane S. P. (2021). High-resolution metabolic mapping of the cerebellum using 2D zoom magnetic resonance spectroscopic imaging. Magn. Reson. Med. 85, 2349–2358. doi: 10.1002/mrm.28614, PMID: 33283917 PMC9307136

[ref42] Estrada-Cuzcano A. Martin S. Chamova T. Synofzik M. Timmann D. Holemans T.. (2017). Loss-of-function mutations in the ATP13A2/PARK9 gene cause complicated hereditary spastic paraplegia (SPG78). Brain 140, 287–305. doi: 10.1093/brain/aww307, PMID: 28137957 PMC5278306

[ref43] Fahey M. C. Cremer P. D. Aw S. T. Millist L. Todd M. J. White O. B.. (2008). Vestibular, saccadic and fixation abnormalities in genetically confirmed Friedreich ataxia. Brain 131, 1035–1045. doi: 10.1093/brain/awm323, PMID: 18238798

[ref44] Federico A. Gallus G. N. (2022). “Cerebrotendinous Xanthomatosis” in Gene Reviews®. eds. Adam M. P. Feldman J. Mirzaa G. M. Pagon R. A. Wallace S. E. (Seattle (WA): University of Washington) 20301583

[ref45] Federighi P. Ramat S. Rosini F. Pretegiani E. Federico A. Rufa A. (2017). Characteristic eye movements in ataxia-telangiectasia-like disorder: an explanatory hypothesis. Front. Neurol. 8:596. doi: 10.3389/fneur.2017.00596, PMID: 29170652 PMC5684103

[ref46] Fernandez-Capetillo O. Lee A. Nussenzweig M. Nussenzweig A. (2004). H2AX: the histone Guardian of the genome. DNA Repair 3, 959–967. doi: 10.1016/j.dnarep.2004.03.024, PMID: 15279782

[ref47] Ferreirinha F. Quattrini A. Pirozzi M. Valsecchi V. Dina G. Broccoli V.. (2004). Axonal degeneration in Paraplegin-deficient mice is associated with abnormal mitochondria and impairment of axonal transport. J. Clin. Investig. 113, 231–242. doi: 10.1172/JCI200420138, PMID: 14722615 PMC311437

[ref48] Fortuna F. Barboni P. Liguori R. Valentino M. L. Savini G. Gellera C.. (2009). Visual system involvement in patients with Friedreich’s ataxia. Brain 132, 116–123. doi: 10.1093/brain/awn269, PMID: 18931386

[ref49] Freedman S. F. Brennand C. Chiang J. DeBarber A. del Monte M. A. Duell P. B.. (2019). Prevalence of Cerebrotendinous Xanthomatosis among patients diagnosed with acquired juvenile-onset idiopathic bilateral cataracts. JAMA Ophthalmol 137, 1312–1316. doi: 10.1001/jamaophthalmol.2019.3639, PMID: 31536098 PMC6753501

[ref50] Frismand S. Salem H. Panouilleres M. Pélisson D. Jacobs S. Vighetto A.. (2013). MRI findings in AOA2: cerebellar atrophy and abnormal iron detection in dentate nucleus. Neuro Image Clin 2, 542–548. doi: 10.1016/j.nicl.2013.03.018 PMC3777765 24179805

[ref51] Furman J. M. Perlman S. Baloh R. W. (1983). Eye movements in Friedreich’s ataxia. Arch. Neurol. 40, 343–346. doi: 10.1001/archneur.1983.04050060043006 6847438

[ref52] Gabsi S. Gouider-Khouja N. Belal S. Fki M. Kefi M. Turki I.. (2001). Effect of vitamin E supplementation in patients with ataxia with vitamin E deficiency. Eur. J. Neurol. 8, 477–481. doi: 10.1046/j.1468-1331.2001.00273.x, PMID: 11554913

[ref53] Galatolo D. de Michele G. Silvestri G. Leuzzi V. Casali C. Musumeci O.. (2021). Ngs in hereditary ataxia: when rare becomes frequent. Int. J. Mol. Sci. 22:8490. doi: 10.3390/ijms22168490, PMID: 34445196 PMC8395181

[ref54] Garces P. Antoniades C. A. Sobanska A. Kovacs N. Ying S. H. Gupta A. S.. (2023). Quantitative Oculomotor assessment in hereditary ataxia: systematic review and consensus by the ataxia global initiative working group on digital-motor biomarkers. Cerebellum. doi: 10.1007/s12311-023-01559-9, PMID: 37117990 PMC11102387

[ref55] Garcia-Martin E. Pablo L. E. Gazulla J. Polo V. Ferreras A. Larrosa J. M. (2013). Retinal nerve fibre layer thickness in ARSACS: myelination or hypertrophy? Br. J. Ophthalmol. 97, 238–241. doi: 10.1136/bjophthalmol-2012-302309, PMID: 23077228 PMC3582091

[ref56] Gatti R. A. Tward A. Concannon P. (1999). Cancer risk in ATM heterozygotes: a model of phenotypic and mechanistic differences between missense and truncating mutations. Mol. Genet. Metab. 68, 419–423. doi: 10.1006/mgme.1999.2942, PMID: 10607471

[ref57] Gazulla J. Vela A. C. Marín M. A. Pablo L. Santorelli F. M. Benavente I.. (2011). Is the ataxia of Charlevoix-Saguenay a developmental disease? Med. Hypotheses 77, 347–352. doi: 10.1016/j.mehy.2011.05.011, PMID: 21665375

[ref58] Giorgio C. Roberto M.. (2018). Spastic paraplegia 7. GeneReviews®. Eds. Adam M. P.. Seattle (WA): University of Washington.

[ref59] Girard M. Larivière R. Parfitt D. A. Deane E. C. Gaudet R. Nossova N.. (2012). Mitochondrial dysfunction and Purkinje cell loss in autosomal recessive spastic ataxia of Charlevoix-Saguenay (ARSACS). Proc. Natl. Acad. Sci. U. S. A. 109, 1661–1666. doi: 10.1073/pnas.1113166109, PMID: 22307627 PMC3277168

[ref60] Guillain G. Bertrand I. Godet-Guillain J. (1942). Etude Anatomo-Clinique d’un Cas de Cholestérinose Cérébrale. Arch Neurol 74, 249–263.

[ref61] Harding A. E. (1981). FRIEDREICH’S ataxia: a clinical and genetic study of 90 families with an analysis of early diagnostic CRITERIA and INTRAFAMILIAL clustering of clinical features. Brain 104, 589–620. doi: 10.1093/brain/104.3.589, PMID: 7272714

[ref62] Hewamadduma C. A. Hoggard N. O’Malley R. Robinson M. K. Beauchamp N. J. Segamogaite R.. (2018). Novel genotype-phenotype and MRI correlations in a large cohort of patients with SPG7 mutations. Neurol Genet 4:e279. doi: 10.1212/NXG.0000000000000279, PMID: 30533525 PMC6244025

[ref63] Hocking D. R. Fielding J. Corben L. A. Cremer P. D. Millist L. White O. B.. (2010). Ocular motor fixation deficits in Friedreich ataxia. Cerebellum 9, 411–418. doi: 10.1007/s12311-010-0178-5, PMID: 20467851

[ref64] Horvath R. Czermin B. Gulati S. Demuth S. Houge G. Pyle A.. (2012). Adult-onset cerebellar ataxia due to mutations in CABC1/ADCK3. J. Neurol. Neurosurg. Psychiatry 83, 174–178. doi: 10.1136/jnnp-2011-301258, PMID: 22036850

[ref65] Imrie J. Dasgupta S. Besley G. T. N. Harris C. Heptinstall L. Knight S.. (2007). The natural history of Niemann–pick disease type C in the UK. J. Inherit. Metab. Dis. 30, 51–59. doi: 10.1007/s10545-006-0384-7, PMID: 17160617

[ref66] Iwasa K. Shima K. Komai K. Nishida Y. Yokota T. Yamada M. (2014). Retinitis Pigmentosa and macular degeneration in a patient with ataxia with isolated vitamin e deficiency with a novel c.717 Del C mutation in the TTPA gene. J. Neurol. Sci. 345, 228–230. doi: 10.1016/j.jns.2014.07.001, PMID: 25066259

[ref67] Kapoor R. Walters S. P. Al-Aswad L. A. (2019). The current state of artificial intelligence in ophthalmology. Surv. Ophthalmol. 64, 233–240. doi: 10.1016/j.survophthal.2018.09.002 30248307

[ref68] Kemp K. C. Cook A. J. Redondo J. Kurian K. M. Scolding N. J. Wilkins A. (2016). Purkinje cell injury, structural plasticity and fusion in patients with Friedreich’s ataxia. Acta Neuropathol. Commun. 4:53. doi: 10.1186/s40478-016-0326-3, PMID: 27215193 PMC4877974

[ref69] Khan A. O. Aldahmesh M. A. Mohamed J. Y. Alkuraya F. S. (2013). Juvenile cataract morphology in 3 siblings not yet diagnosed with Cerebrotendinous Xanthomatosis. Ophthalmology 120, 956–960. doi: 10.1016/j.ophtha.2012.10.032, PMID: 23375591

[ref70] Kipli K. Hoque M. E. Lim L. T. Mahmood M. H. Sahari S. K. Sapawi R.. (2018). A review on the extraction of quantitative retinal microvascular image feature. Comput. Math. Methods Med. 2018, 1–21. doi: 10.1155/2018/4019538, PMID: 30065780 PMC6051289

[ref71] Klebe S. Depienne C. Gerber S. Challe G. Anheim M. Charles P.. (2012). Spastic paraplegia gene 7 in patients with spasticity and/or optic neuropathy. Brain 135, 2980–2993. doi: 10.1093/brain/aws240, PMID: 23065789 PMC3470714

[ref72] Koyama S. Sekijima Y. Ogura M. Hori M. Matsuki K. Miida T.. (2021). Cerebrotendinous Xanthomatosis: molecular pathogenesis, clinical Spectrum, diagnosis, and disease-modifying treatments. J. Atheroscler. Thromb. 28, 905–925. doi: 10.5551/jat.RV17055, PMID: 33967188 PMC8532057

[ref73] Kuriyama M. Fujiyama J. Yoshidome H. Takenaga S. Matsumuro K. Kasama T.. (1991). Cerebrotendinous Xanthomatosis: clinical and biochemical evaluation of eight patients and review of the literature. J. Neurol. Sci. 102, 225–232. doi: 10.1016/0022-510X(91)90073-G, PMID: 2072121

[ref74] Lax N. Z. Hepplewhite P. D. Reeve A. K. Nesbitt V. McFarland R. Jaros E.. (2012). Cerebellar ataxia in patients with mitochondrial DNA disease. J. Neuropathol. Exp. Neurol. 71, 148–161. doi: 10.1097/NEN.0b013e318244477d, PMID: 22249460 PMC3272439

[ref75] Le Ber I. Moreira M. C. Rivaud-Péchoux S. Chamayou C. Ochsner F. Kuntzer T.. (2003). Cerebellar ataxia with Oculomotor apraxia type 1: clinical and genetic studies. Brain 126, 2761–2772. doi: 10.1093/brain/awg283 14506070

[ref76] Leigh R. J. Zee D. S. (2015). The neurology of eye movements. United Kingdom: Oxford University Press.

[ref77] Lewis R. F. Lederman H. M. Crawford T. O. (1999). Ocular motor abnormalities in ataxia telangiectasia. Ann. Neurol. 46, 287–295. doi: 10.1002/1531-8249(199909)46:3<287::AID-ANA3>3.0.CO;2-0, PMID: 10482258

[ref78] Li X. Fan F. Chen X. Li J. Ning L. Lin K.. (2021). Computer vision for brain disorders based primarily on ocular responses. Front. Neurol. 12:4270. doi: 10.3389/fneur.2021.584270 PMC8096911 33967931

[ref79] Mariotti C. Gellera C. Rimoldi M. Mineri R. Uziel G. Zorzi G.. (2004). Ataxia with isolated vitamin E deficiency: neurological phenotype, clinical follow-up and novel mutations in TTPA gene in Italian families. Neurol. Sci. 25, 130–137. doi: 10.1007/s10072-004-0246-z, PMID: 15300460

[ref80] Martelli A. Puccio H. (2014). Dysregulation of cellular iron metabolism in Friedreich ataxia: from primary iron-sulfur cluster deficit to mitochondrial iron accumulation. Front Pharmacol 5:130. doi: 10.3389/fphar.2014.00130 24917819 PMC4042101

[ref81] Martinelli P. Rugarli E. I. (2010). Emerging roles of mitochondrial proteases in Neurodegeneration. Biochim Biophys Acta 1797, 1:10. doi: 10.1016/j.bbabio.2009.07.013 19664590

[ref82] Maudoux A. Teissier N. Francois M. van den Abbeele T. Alberti C. Husson I.. (2020). Vestibular impact of Friedreich ataxia in early onset patients. Cerebell Ataxias 7:6. doi: 10.1186/s40673-020-00115-z, PMID: 32514364 PMC7254732

[ref83] McKenna P. Morgan S. J. Bosanquet R. C. Laker M. F. (1990). A case of Cerebrotendinous Xanthomatosis II: the sterol content of a Cataractous lens. Br. J. Ophthalmol. 74, 629–630. doi: 10.1136/bjo.74.10.629 2285688 PMC1042235

[ref84] Mignarri A. Gallus G. N. Dotti M. T. Antonio F. (2014). A suspicion index for early diagnosis and treatment of Cerebrotendinous Xanthomatosis. J. Inherit. Metab. Dis. 37, 421–429. doi: 10.1007/s10545-013-9674-3 24442603

[ref85] Milea D. Najjar R. P. Zhubo J. Ting D. Vasseneix C. Xu X.. (2020). Artificial intelligence to detect papilledema from ocular fundus photographs. N. Engl. J. Med. 382, 1687–1695. doi: 10.1056/NEJMoa1917130, PMID: 32286748

[ref86] Minakaran N. de Carvalho E. R. Petzold A. Wong S. H. (2021). Optical coherence tomography (OCT) in Neuro-ophthalmology. Eye 35, 17–32. doi: 10.1038/s41433-020-01288-x 33239763 PMC7852683

[ref87] Minnerop M. Kurzwelly D. Wagner H. Soehn A. S. Reichbauer J. Tao F.. (2017). Hypomorphic mutations in POLR3A are a frequent cause of sporadic and recessive spastic ataxia. Brain 140, 1561–1578. doi: 10.1093/brain/awx095, PMID: 28459997 PMC6402316

[ref88] Morani F. Doccini S. Sirica R. Paterno M. Pezzini F. Ricca I.. (2019). Functional Transcriptome analysis in ARSACS KO cell model reveals a role of Sacsin in autophagy. Sci. Rep. 9:11878. doi: 10.1038/s41598-019-48047-x, PMID: 31417125 PMC6695435

[ref89] Moreira M.-C. Klur S. Watanabe M. Németh A. H. Ber I. L. Moniz J.-C.. (2004). Senataxin, the Ortholog of a yeast RNA helicase, is mutant in ataxia-ocular apraxia 2. Nat. Genet. 36, 225–227. doi: 10.1038/ng1303, PMID: 14770181

[ref90] Morgan S. J. McKenna P. Bosanquet R. C. (1989). Case of Cerebrotendinous Xanthomatosis. I: unusual ophthalmic features. Br. J. Ophthalmol. 73, 1011–1014. 2611184 10.1136/bjo.73.12.1011 PMC1041958

[ref91] Naess K. Barbaro M. Bruhn H. Wibom R. Nennesmo I. von Döbeln U.. (2011). Complete deletion of a POLG1 allele in a patient with Alpers syndrome. JIMD Rep 4, 67–73. doi: 10.1007/8904_2011_73 23430898 PMC3509876

[ref92] Naureckiene S. Sleat D. E. Lackland H. Fensom A. Vanier M. T. Wattiaux R.. (2000). Identification of HE1 as the second gene of Niemann-pick C disease. Science 290, 2298–2301. doi: 10.1126/science.290.5500.2298, PMID: 11125141

[ref93] Newman N. J. Yu-Wai-Man P. Biousse V. Carelli V. (2023). Understanding the molecular basis and pathogenesis of hereditary optic neuropathies: towards improved diagnosis and management. Lancet Neurol 22, 172–188. doi: 10.1016/S1474-4422(22)00174-0 36155660

[ref94] Noval S. Contreras I. Sanz-Gallego I. Manrique R. K. Arpa J. (2012). Ophthalmic features of Friedreich ataxia. Eye 26, 315–320. doi: 10.1038/eye.2011.291, PMID: 22094302 PMC3272198

[ref95] Palmeri S. Rufa A. Pucci B. Santarnecchi E. Malandrini A. Stromillo M. L.. (2013). Clinical course of two Italian siblings with ataxia-telangiectasia-like disorder. Cerebellum 12, 596–599. doi: 10.1007/s12311-013-0460-4, PMID: 23436002

[ref96] Panwar N. Huang P. Lee J. Keane P. A. Chuan T. S. Richhariya A.. (2016). Fundus photography in the 21st century-a review of recent technological advances and their implications for worldwide healthcare. Telemed e-Health 22, 198–208. doi: 10.1089/tmj.2015.0068, PMID: 26308281 PMC4790203

[ref97] Parkinson M. H. Bartmann A. P. Clayton L. M. S. Nethisinghe S. Pfundt R. Chapple J. P.. (2018). Optical coherence tomography in autosomal recessive spastic ataxia of Charlevoix-Saguenay. Brain 141, 989–999. doi: 10.1093/brain/awy028, PMID: 29538656

[ref98] Patterson M. (2020). “Niemann-pick disease type C” in GeneReviews®. eds. Adam M. P. Feldman J. Mirzaa G. M.. (Seattle (WA): University of Washington) 20301473

[ref99] Patterson M. C. Clayton P. Gissen P. Anheim M. Bauer P. Bonnot O.. (2017). Recommendations for the detection and diagnosis of Niemann-pick disease type C. Neurology 7, 499–511. doi: 10.1212/CPJ.0000000000000399 29431164 PMC5800709

[ref100] Pfeffer G. Gorman G. S. Griffin H. Kurzawa-Akanbi M. Blakely E. L. Wilson I.. (2014). Mutations in the SPG7 gene cause chronic progressive external Ophthalmoplegia through disordered mitochondrial DNA maintenance. Brain 137, 1323–1336. doi: 10.1093/brain/awu060, PMID: 24727571 PMC3999722

[ref101] Pujari A. Bhaskaran K. Sharma P. Singh P. Phuljhele S. Saxena R.. (2021). Optical coherence tomography angiography in Neuro-ophthalmology: current clinical role and future perspectives. Surv. Ophthalmol. 66, 471–481. doi: 10.1016/j.survophthal.2020.10.009, PMID: 33157113

[ref102] Rahman S. Copeland W. C. (2019). POLG-related disorders and their neurological manifestations. Nat. Rev. Neurol. 15, 40–52. doi: 10.1038/s41582-018-0101-0 30451971 PMC8796686

[ref103] Rajalakshmi R. Prathiba V. Arulmalar S. Usha M. (2021). Review of retinal cameras for global coverage of diabetic retinopathy screening. Eye 35, 162–172. doi: 10.1038/s41433-020-01262-7 33168977 PMC7852572

[ref104] Rezende Filho F. M. Bremner F. Pedroso J. L. de Andrade J. B. C. Marianelli B. F. Lourenço C. M.. (2021). Retinal architecture in autosomal recessive spastic ataxia of Charlevoix-Saguenay (ARSACS): insights into disease pathogenesis and biomarkers. Mov. Disord. 36, 2027–2035. doi: 10.1002/mds.28612 33893680

[ref105] Richard P. Rosonina E. (2021). Regulating autophagy: a novel role for SETX (Senataxin). Neural Regen. Res. 16, 2008–2009. doi: 10.4103/1673-5374.308091 33642381 PMC8343329

[ref106] Rojas P. de Hoz R. Cadena M. Salobrar-García E. Fernández-Albarral J. A. López-Cuenca I.. (2021). Neuro-ophthalmological findings in Friedreich’s ataxia. J Personal Med 11:708. doi: 10.3390/jpm11080708, PMID: 34442352 PMC8398238

[ref107] Romano L. E. L. Aw W. Y. Hixson K. M. Novoselova T. V. Havener T. M. Howell S.. (2022). Multi-Omic profiling reveals the ataxia protein Sacsin is required for integrin trafficking and synaptic organization. Cell Rep. 41:111580. doi: 10.1016/j.celrep.2022.111580, PMID: 36323248 PMC9647044

[ref108] Ronsin S. Hannoun S. Thobois S. Petiot P. Vighetto A. Cotton F.. (2019). A new MRI marker of ataxia with Oculomotor apraxia. Eur. J. Radiol. 110, 187–192. doi: 10.1016/j.ejrad.2018.11.035, PMID: 30599859

[ref109] Rosini F. Pretegiani E. Mignarri A. Optican L. M. Serchi V. de Stefano N.. (2017). The role of dentate nuclei in human Oculomotor control: insights from Cerebrotendinous Xanthomatosis. J. Physiol. 595, 3607–3620. doi: 10.1113/JP273670, PMID: 28168705 PMC5451708

[ref110] Rossi M. Anheim M. Durr A. Klein C. Koenig M. Synofzik M.. (2018). The genetic nomenclature of recessive cerebellar ataxias. Mov. Disord. 33, 1056–1076. doi: 10.1002/mds.27415, PMID: 29756227

[ref111] Rouzier C. Chaussenot A. Serre V. Fragaki K. Bannwarth S. Ait-el-Mkadem S.. (2014). Quantitative multiplex PCR of short fluorescent fragments for the detection of large intragenic POLG rearrangements in a large French cohort. Eur. J. Hum. Genet. 22, 542–550. doi: 10.1038/ejhg.2013.171, PMID: 23921535 PMC3953900

[ref112] Safdar A. Annis S. Kraytsberg Y. Laverack C. Saleem A. Popadin K.. (2016). Amelioration of premature aging in MtDNA Mutator mouse by exercise: the interplay of oxidative stress, PGC-1α, P53, and DNA damage. A hypothesis. Curr. Opin. Genet. Dev. 38, 127–132. doi: 10.1016/j.gde.2016.06.011, PMID: 27497229 PMC5592087

[ref113] Salem I. H. Beaudin M. Klein C. J. Dupré N. (2023). Treatment and Management of Autosomal Recessive Cerebellar Ataxias: current advances and future perspectives. CNS Neurol Disord Drug Targets 22, 678–697. doi: 10.2174/1871527321666220418114846 35440322

[ref114] Salen G. (1971). Cholestanol deposition in Cerebrotendinous Xanthomatosis. A possible mechanism. Ann. Intern. Med. 75, 843–851. doi: 10.7326/0003-4819-75-6-843 5134895

[ref115] Salen G. Steiner R. D. (2017). Epidemiology, diagnosis, and treatment of Cerebrotendinous Xanthomatosis (CTX). J. Inherit. Metab. Dis. 40, 771–781. doi: 10.1007/s10545-017-0093-8 28980151

[ref116] Salviati L. Trevisson E. Rodriguez Hernandez M. A. Casarin A. Pertegato V. Doimo M.. (2012). Haploinsufficiency of COQ4 causes coenzyme Q10 deficiency. J. Med. Genet. 49, 187–191. doi: 10.1136/jmedgenet-2011-100394, PMID: 22368301 PMC3983946

[ref117] Sánchez-Ferrero E. Coto E. Beetz C. Gámez J. Corao A. I. Díaz M.. (2013). SPG7 mutational screening in spastic paraplegia patients supports a dominant effect for some mutations and a pathogenic role for p.A510V. Clin. Genet. 83, 257–262. doi: 10.1111/j.1399-0004.2012.01896.x, PMID: 22571692

[ref118] Satya-Murti S. Howard L. Krohel G. Wolf B. (1986). The Spectrum of neurologic disorder from vitamin E deficiency. Neurology 36, 917–921. doi: 10.1212/wnl.36.7.917 3714053

[ref119] Schuelke M. (2023). “Ataxia with vitamin E deficiency” in GeneReviews®. eds. Adam M. P.. (Seattle (WA): University of Washington) 20301419

[ref120] Seyer L. A. Galetta K. Wilson J. Sakai R. Perlman S. Mathews K.. (2013). Analysis of the visual system in Friedreich ataxia. J. Neurol. 260, 2362–2369. doi: 10.1007/s00415-013-6978-z, PMID: 23775342

[ref121] Shaikh A. G. Marti S. Tarnutzer A. A. Palla A. Crawford T. O. Straumann D.. (2011). Ataxia telangiectasia: a ‘disease model’ to understand the cerebellar control of vestibular reflexes. J. Neurophysiol. 105, 3034–3041. doi: 10.1152/jn.00721.2010, PMID: 21471399

[ref122] Shaikh A. G. Marti S. Tarnutzer A. A. Palla A. Crawford T. O. Straumann D.. (2009). Gaze fixation deficits and their implication in ataxia-telangiectasia. J. Neurol. Neurosurg. Psychiatry 80, 858–864. doi: 10.1136/jnnp.2008.170522, PMID: 19357126

[ref123] Stelten B. M. L. Huidekoper H. H. van de Warrenburg B. P. C. Brilstra E. H. Hollak C. E. M. Haak H. R.. (2019). Long-term treatment effect in Cerebrotendinous Xanthomatosis depends on age at treatment start. Neurology 92, e83–e95. doi: 10.1212/WNL.0000000000006731, PMID: 30530799

[ref124] Stevens J. C. Murphy S. M. Davagnanam I. Phadke R. Anderson G. Nethisinghe S.. (2013). The ARSACS phenotype can include Supranuclear gaze palsy and skin Lipofuscin deposits. J. Neurol. Neurosurg. Psychiatry 84, 114–116. doi: 10.1136/jnnp-2012-303634, PMID: 23123642

[ref125] Stewart G. S. Maser R. S. Stankovic T. Bressan D. A. Kaplan M. I. Jaspers N. G. J.. (1999). The DNA double-Strand break repair gene HMRE11 is mutated in individuals with an ataxia-telangiectasia-like disorder. Cell 99, 577–587. doi: 10.1016/S0092-8674(00)81547-0, PMID: 10612394

[ref126] Stoean C. Stoean R. Atencia M. Abdar M. Velázquez-Pérez L. Khosravi A.. (2020). Automated detection of Presymptomatic conditions in Spinocerebellar ataxia type 2 using Monte Carlo dropout and deep neural network techniques with Electrooculogram signals. Sensor 20:3032. doi: 10.3390/s20113032, PMID: 32471077 PMC7309035

[ref127] Synofzik M. Hélène P. Fanny M. Ludger S. (2019). Autosomal recessive cerebellar ataxias: paving the way toward targeted molecular therapies. Neuron 101, 560–583. doi: 10.1016/j.neuron.2019.01.049 30790538

[ref128] Synofzik M. Schüle R. (2017). Overcoming the divide between ataxias and spastic paraplegias: shared phenotypes, genes, and pathways. Mov. Disord. 32, 332–345. doi: 10.1002/mds.26944 28195350 PMC6287914

[ref129] Synofzik M. Soehn A. S. Gburek-Augustat J. Schicks J. Karle K. N. Schüle R.. (2013). Autosomal recessive spastic ataxia of Charlevoix Saguenay (ARSACS): expanding the genetic, clinical and imaging Spectrum. Orphanet J. Rare Dis. 8:41. doi: 10.1186/1750-1172-8-41, PMID: 23497566 PMC3610264

[ref130] Synofzik M. Srulijes K. Godau J. Berg D. Schöls L. (2012). Characterizing POLG ataxia: clinics, electrophysiology and imaging. Cerebellum 11, 1002–1011. doi: 10.1007/s12311-012-0378-2, PMID: 22528963

[ref131] Szmulewicz D. J. Roberts L. McLean C. A. MacDougall H. G. Halmagyi G. M. Storey E. (2016). Proposed diagnostic Criteria for cerebellar ataxia with neuropathy and vestibular Areflexia syndrome (CANVAS). Neurology 6, 61–68. doi: 10.1212/CPJ.0000000000000215, PMID: 26918204 PMC4753833

[ref132] Tang S. Y. Shaikh A. G. (2019). Past and present of eye movement abnormalities in ataxia-telangiectasia. Cerebellum 18, 556–564. doi: 10.1007/s12311-018-0990-x 30523550 PMC6751135

[ref133] Terryn J. Van Eesbeeck A. Vermeer S. Vandenberghe W. (2020). The characteristic eye movement disorder of RFC1-linked CANVAS. Move Disord Clin Pract 7, 230–231. doi: 10.1002/mdc3.12896 PMC7011655 33636721

[ref134] Thal D. R. Züchner S. Gierer S. Schulte C. Schöls L. Schüle R.. (2015). Abnormal Paraplegin expression in swollen Neurites, τ-and α-Synuclein pathology in a case of hereditary spastic paraplegia SPG7 with an Ala510Val mutation. Int. J. Mol. Sci. 16, 25050–25066. doi: 10.3390/ijms161025050, PMID: 26506339 PMC4632789

[ref135] Thomas-Black G. Altmann D. R. Crook H. Solanky N. Carrasco F. P. Battiston M.. (2023). Multimodal analysis of the visual pathways in Friedreich’s ataxia reveals novel biomarkers. Mov. Disord. 38, 959–969. doi: 10.1002/mds.29277, PMID: 36433650

[ref136] Tibrewal S. Barton Duell P. DeBarber A. E. Loh A. R. (2017). Cerebrotendinous Xanthomatosis: early diagnosis on the basis of juvenile cataracts. J. AAPOS 21, 505–507. doi: 10.1016/j.jaapos.2017.07.211 29079218

[ref137] Tilikete C. Virginie D. (2017). Hypertrophic Olivary degeneration and palatal or Oculopalatal tremor. Front. Neurol. 8:302. doi: 10.3389/fneur.2017.00302 28706504 PMC5490180

[ref138] Traschütz A. Cortese A. Reich S. Dominik N. Faber J. Jacobi H.. (2021). Natural history, phenotypic Spectrum, and discriminative features of multisystemic RFC1 disease. Neurology 96, e1369–e1382. doi: 10.1212/WNL.0000000000011528, PMID: 33495376 PMC8055326

[ref139] Traschütz A. Schirinzi T. Laugwitz L. Murray N. H. Bingman C. A. Reich S.. (2020). Clinico-genetic, imaging and molecular delineation of COQ8A-ataxia: a multicenter study of 59 patients. Ann. Neurol. 88, 251–263. doi: 10.1002/ana.25751, PMID: 32337771 PMC7877690

[ref140] Tulli S. del Bondio A. Baderna V. Mazza D. Codazzi F. Pierson T. M.. (2019). Pathogenic variants in the AFG3L2 Proteolytic domain cause SCA28 through Haploinsufficiency and Proteostatic stress-driven OMA1 activation. J. Med. Genet. 56, 499–511. doi: 10.1136/jmedgenet-2018-105766, PMID: 30910913 PMC6678042

[ref141] Van Bogaert L. Scherer H. Epstein E. (1937). Une Forme Cérébrale de La Cholestérinose Généralisée Edn. Masson. Paris.

[ref142] van Gassen K. L. I. van der Heijden C. D. C. C. de Bot S. T. den Dunnen W. F. A. van den Berg L. H. Verschuuren-Bemelmans C. C.. (2012). Genotype-phenotype correlations in spastic paraplegia type 7: a study in a large Dutch cohort. Brain 135, 2994–3004. doi: 10.1093/brain/aws224 22964162

[ref143] Vermeer S. Warrenburg B. P. Kamsteeg E. J. Brais B. Synofzik M. (2020). “ARSACS” in GeneReviews®. eds. Adam M. P. Feldman J. Mirzaa G. M.. (Seattle (WA): University of Washington) 20301432

[ref145] Vill K. Müller-Felber W. Gläser D. Kuhn M. Teusch V. Schreiber H.. (2018). SACS variants are a relevant cause of autosomal recessive hereditary motor and sensory neuropathy. Hum. Genet. 137, 911–919. doi: 10.1007/s00439-018-1952-6 30460542

[ref146] Walterfang M. Macfarlane M. D. Looi J. C. L. Abel L. Bowman E. Fahey M. C.. (2012). Pontine-to-midbrain ratio indexes ocular-motor function and illness stage in adult Niemann-pick disease type C. Eur. J. Neurol. 19, 462–467. doi: 10.1111/j.1468-1331.2011.03545.x, PMID: 22329857

[ref147] Wiethoff S. Ahmad Z. Ludger S. Manuel D. F. (2012). Retinal nerve fibre layer loss in hereditary spastic paraplegias is restricted to complex phenotypes. BMC Neurol. 12:143. doi: 10.1186/1471-2377-12-143 23176075 PMC3564819

[ref148] Wong L. J. C. Naviaux R. K. Brunetti-Pierri N. Zhang Q. Schmitt E. S. Truong C.. (2008). Molecular and clinical genetics of mitochondrial diseases due to POLG mutations. Hum. Mutat. 29, E150–E172. doi: 10.1002/humu.20824, PMID: 18546365 PMC2891192

[ref149] Yokota T. Uchihara T. Kumagai J. Shiojiri T. Pang J. J. Arita M.. (2000). Postmortem study of ataxia with retinitis Pigmentosa by mutation of the alpha-Tocopherol transfer protein gene. J. Neurol. Neurosurg. Psychiatry 68, 521–525. doi: 10.1136/jnnp.68.4.521, PMID: 10727494 PMC1736898

[ref150] Zee D. S. Yee R. D. Singer H. S. (1977). Congenital ocular motor apraxia. Brain 100, 581–599. doi: 10.1093/brain/100.3.581 589433

